# Tephra fall probabilistic hazard maps for VEI 3 and VEI 4 eruptions at Mt. Garibaldi (*Nch’kay’)* volcano, British Columbia, Canada

**DOI:** 10.1186/s13617-026-00173-8

**Published:** 2026-09-17

**Authors:** Sara Osman, Thomas J. Jones, Yannick Le Moigne, Melanie Kelman

**Affiliations:** 1 Lancaster Environment Centre, Lancaster University, Lancaster, UK; 2 Natural Resources Canada, Geological Survey of Canada, Vancouver, BC Canada; 3 Department of Earth Sciences, Simon Fraser University, Burnaby, BC Canada

**Keywords:** Volcanic hazards, Probabilistic hazard assessment, Probability maps, Tephra dispersal, Canadian volcanism, Garibaldi volcanic belt, Canadian cascades

## Abstract

Following explosive volcanic eruptions, hazard from tephra fall can be widespread. Based on the geological record of past eruptions and risk to population and infrastructure, Mt. Garibaldi volcano (*Nch’kay’*) in British Columbia has been assigned the highest threat ranking of any Canadian volcano. However, until this contribution no hazard map exists for the volcano. In our study, we formed a government-university partnership and modelled tephra dispersion for Volcanic Explosivity Index (VEI) 3 and VEI 4 scenarios for Mt. Garibaldi to produce probabilistic tephra fall hazard maps. We found significant tephra impacts (loads ≥ 0.1 kg m^− 2^ which can cause skid risks on roads and disrupt airports) extend over 810 km^2^ for the VEI 3 scenario and 9360 km^2^ for VEI 4 (with 50% probability of exceedance). Tephra loads that may cause infrastructure damage (≥ 1 kg m^− 2^) cover 250 km^2^ and 1670 km^2^ for the VEI 3 and VEI 4 scenarios, respectively. We combined our results into a single tephra ground accumulation hazard map and information brief. This is the first probabilistic volcanic hazard map using well-constrained source parameters for a Canadian volcano. Further work with emergency managers, will build on this map, toward consideration of appropriate mitigation measures.

**Supplementary Information:**

The online version contains supplementary material available at https://doi.org/10.1186/s13617-026-00173-8.

## Introduction

Volcanic hazards are extremely varied and thus generate a wide range of potential impacts. Hazards from explosive eruptions include pyroclastic density currents (PDCs) (e.g. Dellino et al. 2025; Paine and Wadsworth 2025; Zuccarello et al. 2025), ballistic ejecta (e.g. Biensan et al. 2025; Reale Calafino et al. 2025; Sork et al. 2025), volcanic mudflows or lahars (e.g. Ersöz et al. 2024; Phillips et al. 2024; Cruz-Vázquez et al. 2025), and tephra (e.g. Wilson et al. 2015; Mastin et al. 2021; Paredes-Mariño et al. 2022). Here, we focus on the latter – the production of pyroclasts or tephra which occurs through magma fragmentation during all explosive eruptions (Gonnermann 2015; Jones et al. 2022a).

Such explosive eruptions are capable of producing volcanic ash clouds which can impact aircraft and lead to airspace closure (e.g. Kristiansen et al. 2024; Delbrel et al. 2025; Horwell et al. 2025; Hagenbourger et al. 2026), airborne tephra which can exacerbate respiratory issues (e.g. Covey et al. 2021; Fernandez et al. 2023; Horwell et al. 2024) and ground tephra fallout, where impacts can extend to hundreds of kilometres from the source for the largest eruptions (e.g. Bonadonna et al. 2015; Romero et al. 2021; Hayes et al. 2022). Our contribution here focuses on ground accumulations of tephra. Even thin tephra deposits (< 1 mm thickness) can disrupt transportation (Guffanti et al. 2009; Blake et al. 2017; Mereu et al. 2025; Lerner et al. 2026) and thicker deposits (~ 10 mm) may disrupt power, water and communications networks (e.g. Wilson et al. 2014; Jenkins et al. 2015; Ramírez et al. 2022; Porter et al. 2025), while deposits ~ 100 mm thick or greater may damage buildings (Jenkins et al. 2014; Hayes et al. 2019).

**Fig. 1:**
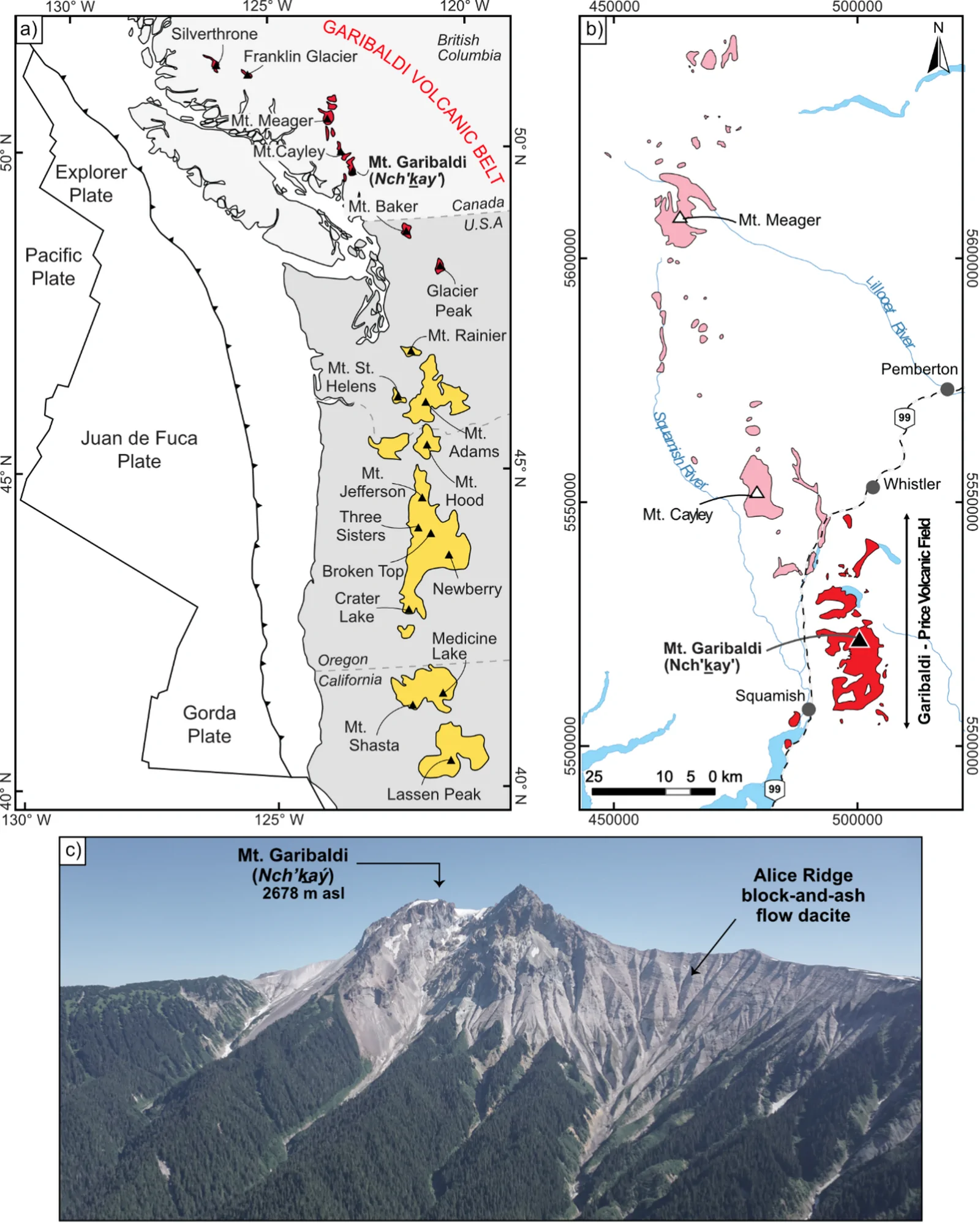
Geological setting, **(a)** Simplified map of the Cascades Volcanic Arc. The Garibaldi Volcanic Belt is the northern extent of the Cascades Volcanic Arc **(b)** The Garibaldi Volcanic Belt in southwestern British Columbia. Mt. Garibaldi (*Nch’kay’)* is the main stratovolcano of the Garibaldi – Price Volcanic Field, located approximately 60 km north of Vancouver **(c)** Panoramic view of Mt. Garibaldi (*Nch’kay’)*, looking east

Since Canada currently has no official government-issued volcanic hazard maps, in this work we model explosive events to produce scenario-based probabilistic tephra fall hazard maps for Mt. Garibaldi or *Nch’kay’* (pronounced in-ch-KAI) in the language of the Squamish (*S*ḵ*wx̱w*ú*7mesh*) First Nation. Mt. Garibaldi, located in southwest British Columbia (Fig. 1a, b), was selected as the target for this study based on its assessment as the highest threat volcano in Canada (Kelman and Wilson 2024, 2025). When compared with volcanoes in the United States, the threat from Mt. Garibaldi ranks slightly higher than Mt. Baker, WA, which has a published hazard map (Gardner et al. 1995) and experienced unrest in 1975 (Crider et al. 2011).

## Geological and geographic setting

The Cascades Volcanic Arc (CVA) extends for more than 1200 km from northern California to southwestern British Columbia and is attributed to the subduction of the Juan de Fuca and Explorer plates beneath the North American plate (Fig. 1a; Hildreth 2007). In northern Washington State, a change in the arc axis orientation mirrors a shift in the subduction orientation, and this geological transition subdivides the CVA into the High Cascades (from Lassen Peak to Mt. Rainier) and the Garibaldi Volcanic Belt (GVB) to the north (Fig. 1a; Hildreth 2007; Mullen and Weis 2013; Russell et al. 2023).

The GVB includes 9 major volcanic fields, listed from south to north: Glacier Peak, Mt. Baker, Garibaldi – Price, Mt. Cayley, Mt. Meager, Salal Glacier, Bridge River, Franklin Glacier, and Mt. Silverthrone (Fig. 1b; Green et al. 1988; Hildreth 2007). The volcanic deposits in the GVB range in composition from alkaline basalts to rhyodacite (Mathews 1958; Green et al. 1988; Wilson and Russell 2019; Russell et al. 2023). Volcanic activity within the GVB started in Early Pleistocene (Green et al. 1988), with the most recent eruption occurring at Glacier Peak ~ 300 years ago (Beget 1982). Within the Canadian portion of the GVB, the last eruption occurred ~ 2.4 ka ago at Mt. Meager, producing 2–3 km^3^ of rhyodacitic materials (Hickson et al. 1999; Andrews et al. 2014), with ashfall deposits identified as far east as Alberta (Stasiuk and Russell 1990; Jensen et al. 2019).

In British Columbia, the GVB includes three en-echelon north-south volcanic segments including the Garibaldi – Price volcanic field (GPVF) situated approximately 60 km north of Vancouver, between the towns of Squamish and Whistler (Fig. 1b). Mt. Garibaldi (2,678 m above sea level; Fig. 1c) is the most conspicuous volcanic edifice of the GPVF. Its geological history and geomorphology are complex and intimately coupled with the

advance and retreat of Cordilleran Ice Sheets (Mathews 1958; Green et al. 1988; Wilson and Russell 2019; Le Moigne and Kelman 2026). Mt. Garibaldi was intermittently active from the Middle Pleistocene to the Holocene with the last eruption occurring at Opal Cone about 9.5 ka erupting ~ 2.8 km^3^ of high-silica andesitic lava (Green et al. 1988; Le Moigne and Kelman 2026). Compositionally, the erupted lavas at Mt. Garibaldi are dominantly dacitic in composition with evidence of effusive (domes, lava flows) and explosive eruptions. Glacial erosion during the Pleistocene has significantly removed the tephra fall record (Green et al. 1988; Le Moigne and Kelman 2026) and the remote location makes fieldwork very difficult (Russell et al. 2023). Hence, forensic characterization of the past explosive eruptions at Mt. Garibaldi is challenging and extremely limited (Kelman and Wilson 2024).

## Volcanic hazard assessment in Canada

Although there is an extensive literature on volcanoes and volcanic hazards in Canada, only two volcanic hazard maps exist, for Nazko Cone (Hickson et al. 2009) and Mt. Meager (Warwick et al. 2022). Previous volcanological research in Canada focused on volcanoes that clearly pose significant threats (e.g. Mt. Meager and Mt. Garibaldi) and at Nazko Cone, where recent unrest has occurred. Other volcanoes that have attracted interest include *Sii Aks* (Tseax), which is documented in the oral histories of the Nis*g*a’a First Nation (e.g. Williams-Jones et al. 2020; Le Moigne et al. 2022a; b; Jones et al. 2025; Osman et al. 2026a; b) and the Wells Gray-Clearwater volcanic field, which displays numerous examples of glaciovolcanism (e.g. Hickson 1986; Hickson et al. 1995). However, until recently, no systematic approach has guided attempts to prioritize work at specific volcanoes based on the risks and potential impacts.

In order to provide a rational basis to plan for future hazard and risk assessments and monitoring, Kelman and Wilson (2024) used the methodologies for volcanic threat assessment developed by the United States Geological Survey (USGS) as part of the National Volcano Early Warning System (NVEWS; Ewert et al. 2005, 2018; Ewert 2007). Every Canadian volcano was systematically ranked on a series of hazard and exposure factors to produce a numerical threat score, making it possible to compare all of Canada’s volcanoes in a semi-quantitative manner. The hazard factors include volcano type, maximum past explosivity, frequency of major explosive activity, eruption recurrence, and historical unrest. Exposure factors include population close to the volcano and proximity to ground-based infrastructure and aviation. At the time of the original threat ranking, the minimal well-understood information available about Mt. Garibaldi’s geology, coupled with its unequivocal exposure to population centres and infrastructure resulted in a numerical threat score which placed it in the Very High threat category. It has a similar score to Mt. Baker and Glacier Peak, and is in the same threat category as better-known volcanoes such as Mount St. Helens (Ewert et al. 2018; Kelman and Wilson 2024). Full details of this threat ranking methodology are provided in Kelman and Wilson (2024, 2025).

### Ongoing and future hazard assessment

The Canada Safety and Security Program has provided funding for the 2022–2027 Volcano Risk Reduction in Canada (VRRC) project which aims to provide tools for evidence-based decision-making about volcanic hazards and risks in Canada. Together with the UK Research and Innovation (UKRI) Future Leaders Fellowship “Flow and fragmentation of melts and magmas” project, based at Lancaster University, UK, the tephra fall hazard mapping work presented here was conducted as part of a broader goal to improve the understanding of explosive volcanism.

The intent of the VRRC project is to promote a more standardized approach to future hazard and risk assessments, incorporating both international best practices in volcanic hazard and risk assessment and Canada-specific considerations. These considerations include long recurrence intervals, remote locations of most volcanoes (Russell et al. 2023), the location of all volcanoes on Indigenous territories (Jones et al. 2024), and the resources available for volcano risk reduction in Canada. Outputs from the project will provide key public safety partners (including emergency managers, civil decision makers, and geoscientists) with information on the probability, nature, magnitude, and extent of volcanic hazards from the GPVF.

The GPVF, and specifically Mt. Garibaldi, has been selected as the focus for the VRRC project, with the aim of completing a comprehensive hazard and risk assessment, incorporating tephra fall and volcanic flow hazard modelling, lithofacies mapping, and other geological analyses. This tephra accumulation modelling is intended to serve as an example of a comprehensive hazard and risk assessment at a very high threat Canadian volcano and as a template for the development of guidelines for conducting future volcanic hazard assessments in Canada.

## Methods

### Scenario selection

Eruptive scenarios for Mt. Garibaldi were developed based on the geological record of past eruptions. The GPVF is dominated by intermediate and felsic compositions and there is abundant evidence for dome collapse eruptions that generated PDCs, although there are no good estimates for plume height and erupted volumes for these events. There is also a pumice fall deposit in the vicinity of Opal Cone, but its source vent, total volume, and extent are not known because it has been extensively remobilised by glacial processes.

The Volcanic Explosivity Index (VEI) categorises the size of explosive eruptions based on the volume of ejecta and plume height (Newhall and Self 1982). Holocene dacitic eruptions within the Cascades Volcanic Arc include VEI 2 to VEI 6 eruptions at Mount St. Helens (Mullineaux and Crandell 1981; Global Volcanism Program 2025) and eruptions at Glacier Peak (Beget 1983), Mt. Rainier (Sisson and Vallance 2009), Newberry Volcano (Rust and Cashman 2007) and Mt. Spurr (Neal et al. 1995) estimated as VEI 3 or 4 (Global Volcanism Program 2025). Given the composition of most sampled units and the history of dome collapse at Mt. Garibaldi, it is plausible that larger magnitude eruptions (e.g., VEI 4) may occur. Hence VEI 4 (0.1–1 km^3^ of ejecta and 10–25 km plume height) was chosen as the largest magnitude eruptive scenario. Although the likelihood of such a scenario occurring is not known, this probably represents the worst-case tephra fall scenario for explosive eruptions from the GPVF and we therefore chose a VEI 3 eruption (0.01–0.1 km^3^ of ejecta and 3–15 km plume height) as our second scenario.

### Tephra2 and TephraProb modelling

Our simulations used the Matlab package TephraProb (Biass et al. 2016) to provide probabilistic outputs from multiple runs of the Tephra2 tephra dispersion model (Bonadonna et al. 2005; Connor and Connor 2006). Eruption source parameters (ESPs) were selected based on field data and analogue eruptions, with wind data stochastically sampled from a 20-year dataset.

**Fig. 2:**
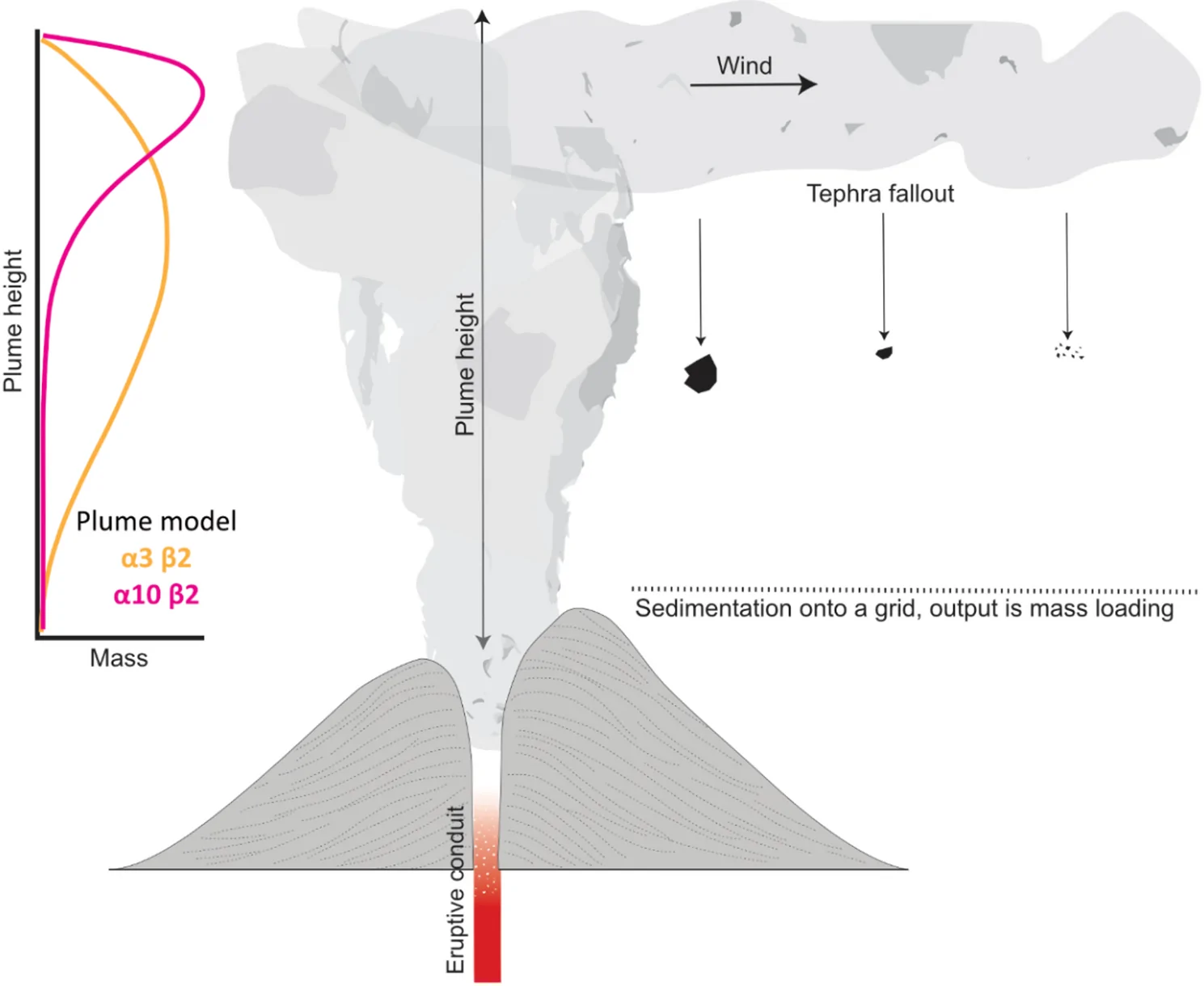
Cartoon showing the main features of a tephra dispersion model (amended from Jones et al. 2022b). Plume model is a β distribution with values of α and β as shown

Tephra2 simulates the release of particles in a volcanic plume and models their transport and sedimentation. The two-dimensional advection-diffusion equation is used to calculate tephra load on the ground (Bonadonna et al. 2005; Connor and Connor 2006). Model inputs include source parameters (plume height, eruption duration and total mass erupted), particle parameters (grain size distribution and particle density) and wind profiles. In addition, the model must be calibrated to select optimum values of empirical model constants that describe the vertical distribution of particles released within the eruption column (defined by a β distribution; Fig. 2) and diffusion within the plume (controlled by the diffusion coefficient and fall time threshold). TephraProb (Biass et al. 2016) provides functionality to run Tephra2 multiple times, with input parameters sampled stochastically, to produce probabilistic tephra load maps.

### Model calibration

Where possible, model calibration uses data from an earlier eruption at the volcano of interest (Bonadonna et al. 2005; Pardini et al. 2023). However, where there are only sparse data on previous eruptions, the model can be calibrated using data from analogue eruptions at volcanoes in similar geological settings, which match the size and composition of the model scenario being considered (e.g. Jenkins et al. 2020; Tennant et al. 2021; Osman et al. 2024). This is the case for Mt. Garibaldi and hence we selected the eruption of Mt. Spurr in September 1992 as an analogue for the VEI 3 scenario and the Mount St. Helens 1980 eruption as a VEI 4 analogue. Both of these past eruptions have sufficient published observations to provide model inputs, including plume height, duration, erupted mass and total grain size distribution, and mapped tephra ground loadings that can be compared to the model output. Details of the calibration are given in Supplementary Material SM1 (Additional File 1).

### Selecting the input total grain size distribution

Total grain size distribution (TGSD) is a key parameter in tephra dispersal models and we followed the method of Costa et al. (2016) to compute an idealised TGSD, using empirical relationships between TGSD, plume height and magma viscosity. The database from which these empirical relationships are derived calculates a 2-phase (solid and melt) magma viscosity considering the phenocrysts only. Thus, for consistency here we do the same.

#### Calculating magma viscosity

In detail, we chose the dacite of Alice Ridge block-and-ash flow deposits (Figs. 1c, S2, Supplementary Material SM2 in Additional File 1) as a representative dacitic composition for Mt. Garibaldi. This unit is located on the southern flank of Atwell Peak and was emplaced during numerous syn-eruptive dome collapses during the late Pleistocene. This block-and-ash flow unit is well preserved, relative to other fragmental deposits of the same age, and has been minimally weathered. The full details of the magma viscosity calculation can be found in Supplementary Material SM2 (Additional File 1). The final 2-phase (i.e., melt and phenocrysts) magma viscosity, from herein simply referred to as the magma viscosity of the Alice Ridge block-and-ash dacite is shown in Table 1. The magma viscosity was calculated using the median bulk rock compositions of fourteen Alice Ridge block-and-ash flow deposit samples, as an approximation of bulk magma composition. The unit is compositionally homogeneous with a median normalized SiO_2_ content of 65.6 wt% (stdev of 0.62 wt%). We then estimated the phenocryst proportions on three representative thin section scans. As the unit is also petrologically homogeneous, we therefore judged these observations to be sufficient to obtain representative phenocryst proportions. The identified phenocryst phases are plagioclase (17.9 vol%), brown amphibole (3.8 vol%), orthopyroxene (2.0 vol%), and minor oxide (0.3 vol%). The compositions of the phenocryst phases were then analysed by Electron Probe Microanalysis (EPMA, see SM2) and the composition of the residual melt was calculated by accounting for the removal of the phenocryst phases and their respective proportions. Following this approach, the calculated pure melt is rhyodacitic in composition with SiO_2_ = 69.2 wt%.

The magma eruption temperature and water content was calculated using the amphibole-only thermometer and hygrometer from Ridolfi and Renzulli (2012). We assumed the amphibole crystallization temperature to be representative of the eruption temperature as indicated by their euhedral shape and absence of developed reaction rims (Rutherford and Devine 2003; De Angelis et al. 2013). The calculated eruption temperature ranges between ~ 820 and ~ 900 °C (mean of 884 °C, and total uncertainty of ± 23 °C), and the dissolved water content at storage conditions ranges between ~ 4.7 and ~ 6.6 wt%. Using the GRD viscosity model (Giordano et al. 2008), we calculated the melt viscosity at these temperatures and for water contents 4 wt% lower than those estimated by the amphibole-only hygrometer to account for magma degassing during ascent (Rutherford et al. 1985; Cashman and Blundy 2000). Finally, we calculated the 2-phase (i.e., melt and crystals) magma viscosity by accounting for the effect of the suspended crystals (i.e., the phenocrysts). We used Equation 2.4 from Mueller et al. (2010) based on the Maron and Pierce (1956) relationship: $\:{\eta\:}_{r}={(1-\:\frac{\phi\:}{{\phi\:}_{m}})}^{-2}$ where *η*_*r*_ = relative apparent viscosity, *φ* = particle volume fraction and *φ*_*m*_ = maximum packing of spherical particles. Table 1 shows the results and the realistic range of magma viscosities expected for future dacitic eruptions at Mt. Garibaldi.

**Table 1:** Bulk viscosity of the Alice Ridge block-and-ash dacite, considered to be representative of Mt. Garibaldi (*Nch’kay*’) dacite magma, calculated using GRD model (Giordano et al. 2008). Values in bold were used for the grain size distribution calculations

Temperature (°C)	H_2_O (wt%)	Viscosity η (Pa s)	log_10_ η (Pa s)
820	0.5	1.74 × 10^8^	**8**
900	0.5	1.25 × 10^7^	7
820	2.5	1.16 × 10^6^	6
900	2.5	1.72 × 10^5^	**5**

#### Calculating a model TGSD

**Fig. 3:**
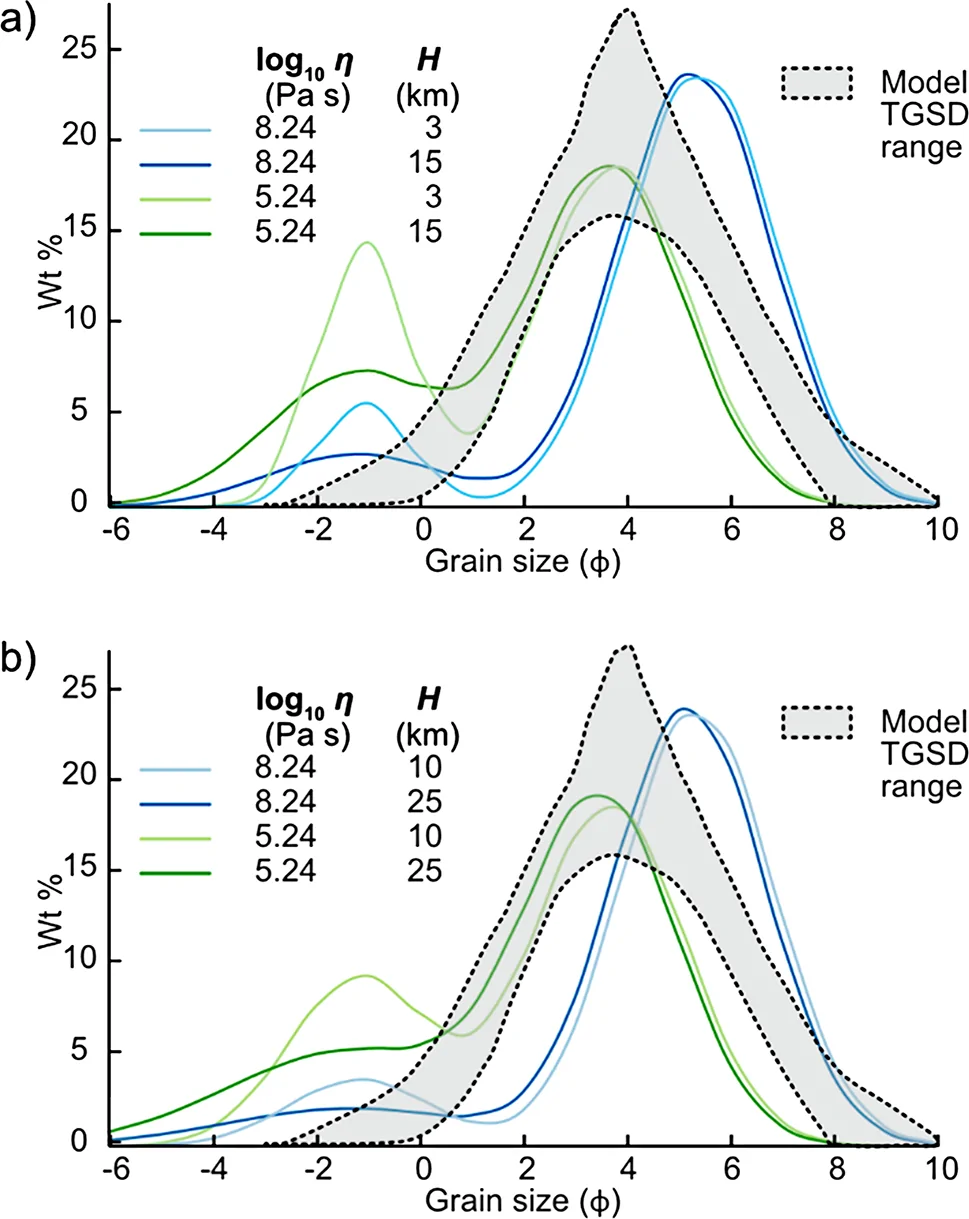
Estimated total grain size distribution (TGSD) for range of plume heights and magma viscosities expected at Mt. Garibaldi (*Nch’kay’)* (using method of Costa et al. 2016). **(a)** VEI 3 eruption; **(b)** VEI 4 eruption. *η* = magma viscosity; *H* = plume height

We took maximum and minimum viscosity values from our calculations (5.24 and 8.24 log_10_ Pa s, respectively; Table 1) and maximum and minimum plume heights for each VEI (3–15 and 10–25 km respectively) as defined in Newhall and Self (1982). We used the method of Costa et al. (2016) to identify four TGSDs which represent likely extremes for a dacitic eruption at Mt. Garibaldi. Each TGSD shows a bimodal grain size distribution, but Tephra2 can only take a unimodal input (Fig. 3). Therefore, by visual inspection, we selected a range of best-fit unimodal TGSDs to use in our model runs (log-normal distributions defined by a range of median and standard deviation values shown in Table 2; Fig. 3).

### Wind data

**Fig. 4:**
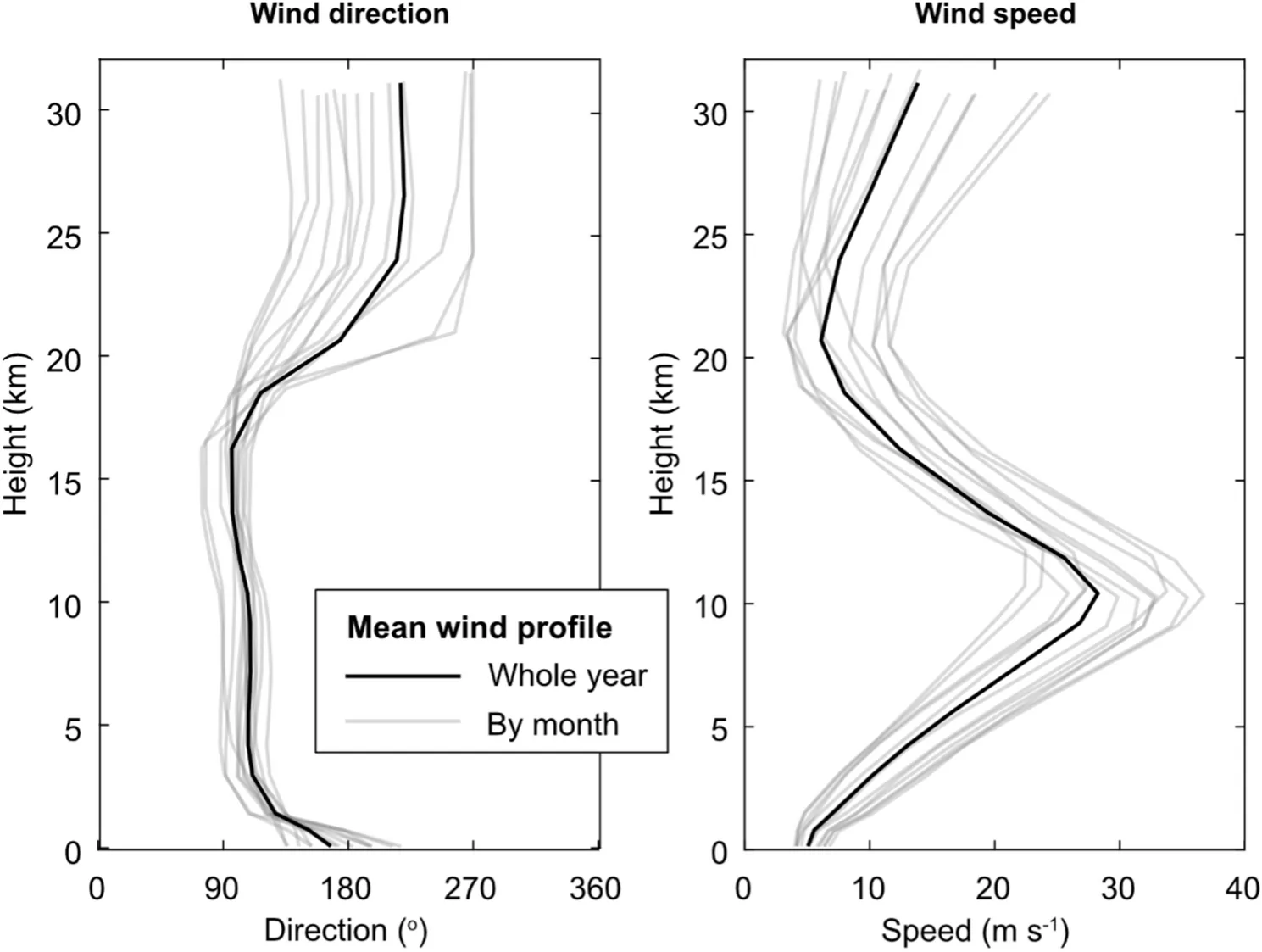
Summary of wind conditions at Mt. Garibaldi (*Nch’kay’)* 2005–2024, from NCEP/DOE Reanalysis II dataset (Kanamitsu et al. 2002). Mean wind speed and mean direction that the wind is blowing towards, by month and for the whole dataset

**Fig. 5:**
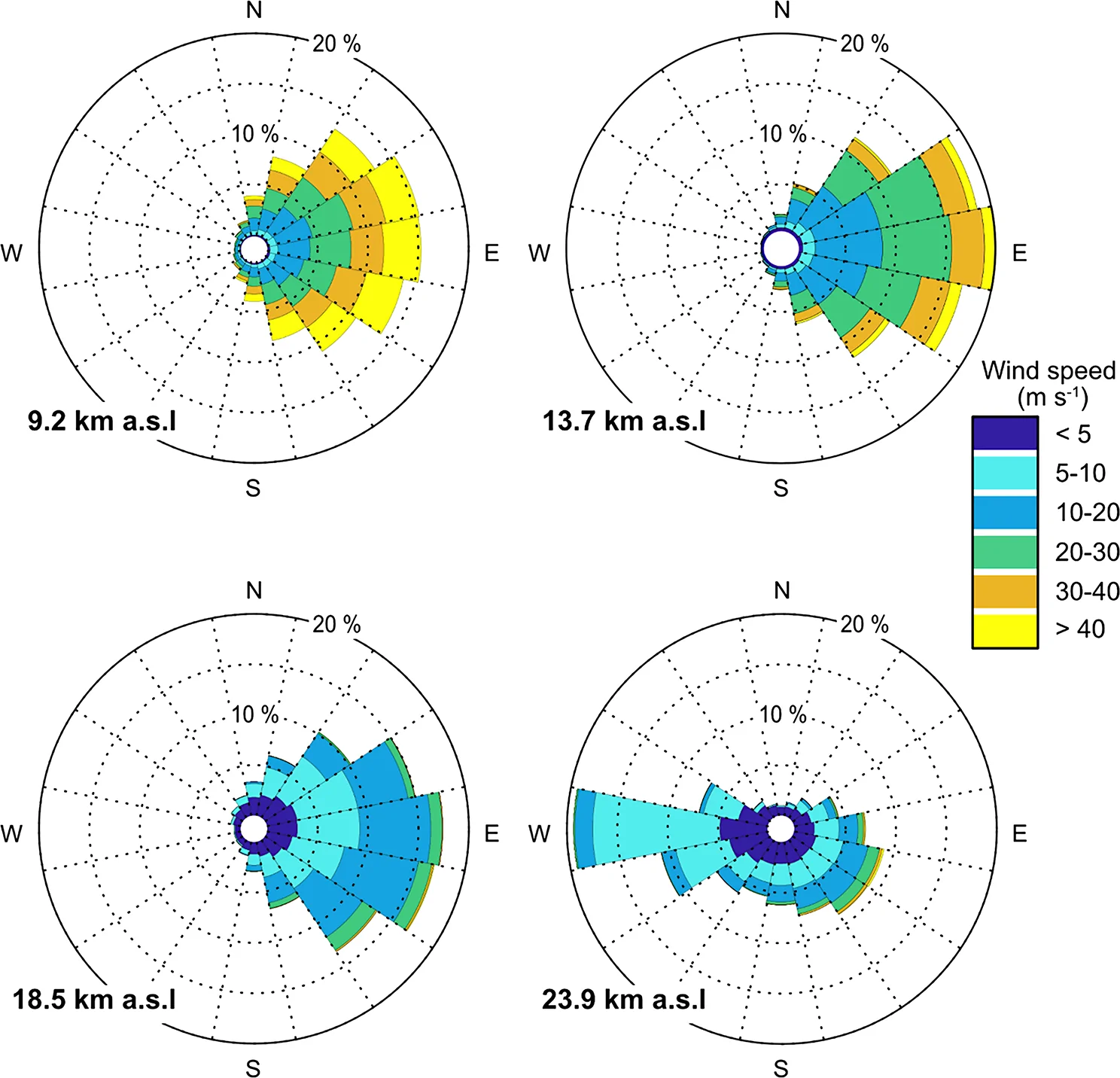
Summary of wind conditions at Mt. Garibaldi (*Nch’kay’)* 2005–2024, from NCEP/DOE Reanalysis II dataset (Kanamitsu et al. 2002). Wind roses for the whole dataset at heights of 9–24 km above sea level (a.s.l.). Wind direction indicates the direction the wind is blowing towards

The wind dataset used in this study comprised 6-hourly NCEP/DOE Reanalysis II data for 2005–2024 (Kanamitsu et al. 2002), a total of 29,220 wind profiles. Each profile included wind velocity data at 17 atmospheric pressure levels. We found no significant seasonal variation in wind speed or direction (Fig. 4), with prevailing winds towards the NE – SE sector, particularly below ~ 20 km above sea level (Fig. 5). Our simulations therefore sampled winds from across the whole dataset, with no subdivisions of month or season.

### Eruption source parameters

For each eruption scenario (i.e., VEI 3 and VEI 4), we ran 10,000 simulations, with the input parameters sampled from the ranges shown in Table 2. Values of the parameters sampled for each run are shown in Supplementary Material SM3 (Additional File 1).

**Table 2:** Input parameters used for both Mt. Garibaldi (*Nch’kay’)* eruption scenarios. Wind profiles are NCEP/NCAR Reanalysis II data provided by NOAA PSL from their website at https://psl.noaa.gov

Parameter	Values		Comments
**Volcano**			
Vent zone	UTM 10 N		
Vent easting, northing	499,662, 5,522,020		
Vent height (m a.s.l.)	2678		
**Computational grid**			
Resolution (m)	2000		
Mean elevation (m a.s.l.)	1026		
**Source parameters**	**VEI 3**	**VEI 4**	
Plume height (km a.s.l.)	3–15	10–25	Plume heights from Newhall and Self (Newhall and Self 1982)
Sampling	Logarithmic	Logarithmic	
Erupted mass (kg)	5 × 10^9^ – 10^11^	5 × 10^10^ – 10^12^	VEI 3 = 0.01–0.1 km^3^; VEI 4 = 0.1–1 km^3^ (Newhall and Self 1982) Dacite bulk density 400–1250 kg m^-3^ (Osman et al. 2022)
Eruption duration (h)	3–9	6–12	
**Grain size and density**			
Max and min (Φ)	-5–9		See Selecting the input total grain size distribution section
Median (Φ)	3.5–4.5		
σ (Φ)	1.5–2.5		
Aggregation coefficient (0–1)	0.2–0.8		Aggregation of particles ≥ 5 Φ
Lithic density (kg m^-3^)	2500		Based on Mount St. Helens dacite (Bonadonna and Phillips 2003)
**Wind profiles**			
Wind dataset	01-Jan-2005–31 Dec 2024		NCEP/NCAR Reanalysis II 6-hourly data (Kanamitsu et al. 2002)
Wind direction (° clockwise from N)	0–360		
Troposphere height (km a.s.l.)	10		(Liu et al. 2014)
**Model constants**			
Eddy constant (m^2^ s^-1^)	0.04		(Connor et al. 2011)
Column integration steps	100		
Particle integration steps	100		
Number of runs	10,000		
Diffusion coefficient (DC; m^2^ s^-1^)	7500		DC, FTT and α, β values taken from model calibration runs:
Fall time threshold (FTT; s)	9700		Mount Spurr (VEI 3)
	**VEI 3**	**VEI 4**	Mount St. Helens (VEI 4)
α, β values of β distribution	10, 2	3, 2	

## Results

We present our results in two ways. First, as probabilities of tephra load exceeding certain thresholds (Table 3). This format is useful when considering how different infrastructure may be affected. Our load thresholds were selected based on likely impacts (Wallace et al. 2001; Jenkins et al. 2014; Blake et al. 2017; Lerner et al. 2026). Second, as tephra loads at fixed probabilities of exceedance (10%, 50% and 90%). This format allows tephra loadings to be assessed over the entire area of interest.

**Table 3:** Tephra load thresholds used to map model outputs and corresponding likely impacts (based on Wallace et al. 2001; Wilson et al. 2012; Leonard et al. 2014; Jenkins et al. 2014, 2015; Blake et al. 2017; Ligot et al. 2022; Ramírez et al. 2022; Lerner et al. 2026)

Tephra load (kg m^− 2^)	Likely impact
0.1	Skid risks on roads, airports
1	Disruption to road, rail, airports Damage to hydropower components, depending on size of catchment area Clogging of air intake to heating, ventilation and air conditioning systems
10	Damage to water treatment pumps by ash in intake water Failure of power and telecommunications cables and flashover on high voltage lines Severe impact to roads and closure of airports Impact on crops, depending on the stage in the growth cycle
100	Collapse of weakest roofs and long span roofs

### Mt. Garibaldi VEI 3 scenario

**Fig. 6:**
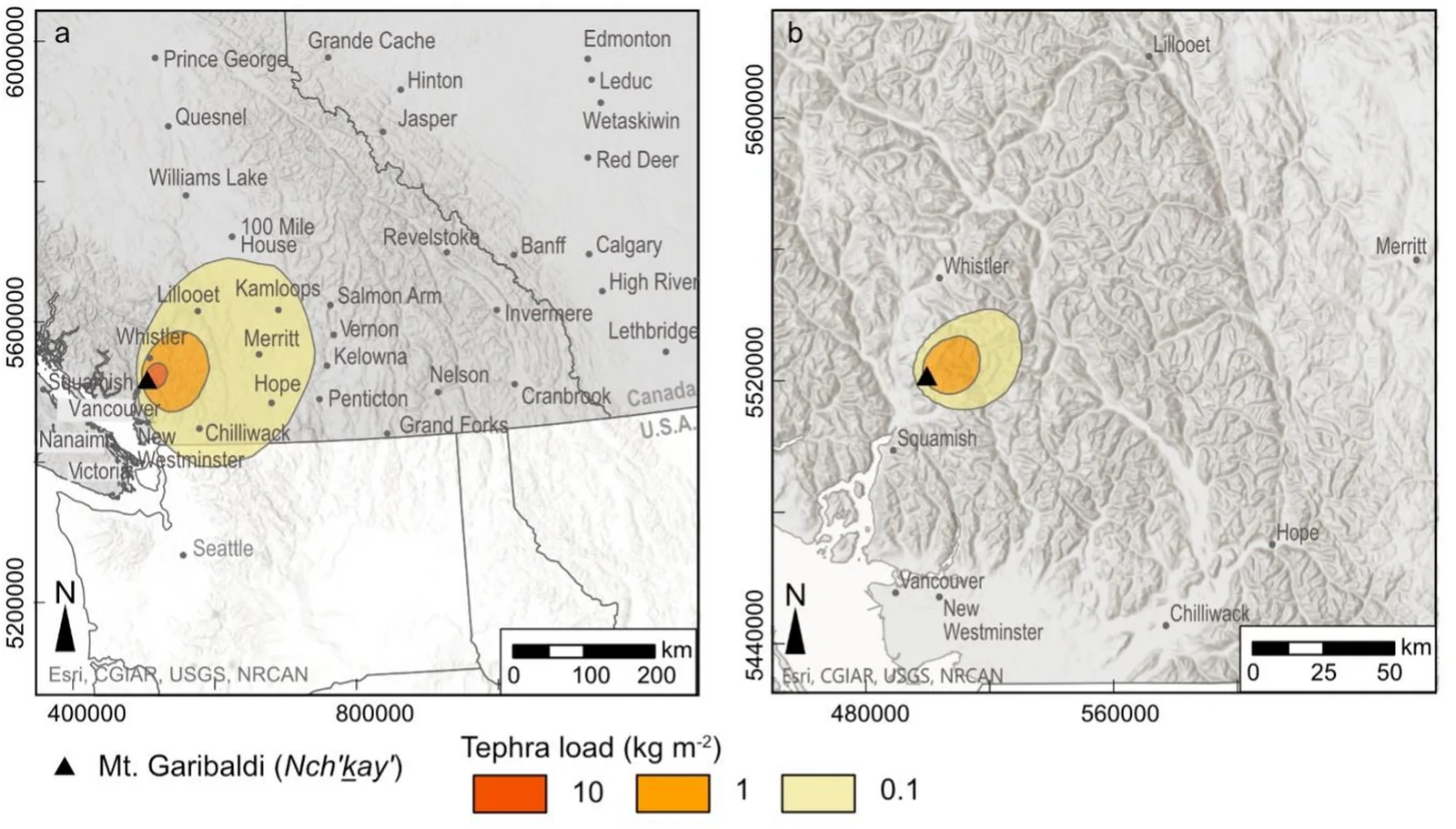
Tephra ground loads from a modelled VEI 3 eruption of Mt. Garibaldi (*Nch’kay’)* with probability of exceedance of **(a)** 10% and **(b)** 50%. Coordinate system UTM 10 N, NAD83 Datum. Note: different scales for each map. Area for 90% probability of exceedance is too small to represent

**Fig. 7:**
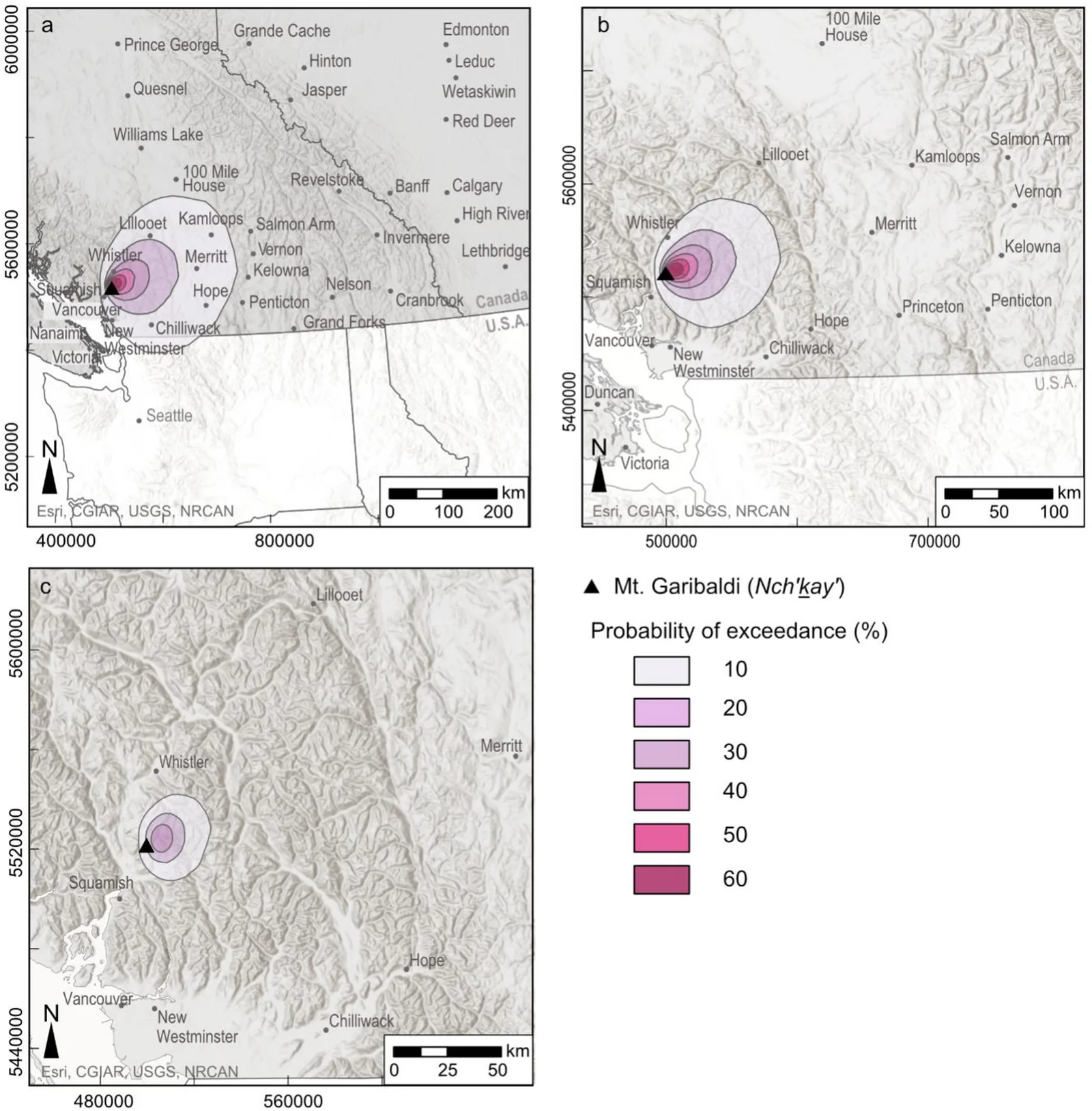
Probability of tephra ground loads from a modelled VEI 3 eruption of Mt. Garibaldi (*Nch’kay’)* exceeding thresholds of **(a)** 0.1 kg m^-2^, **(b)** 1 kg m^-2^ and **(c)** 10 kg m^-2^. Coordinate system UTM 10 N, NAD83 Datum. Note: different scales for each map. Area for 100 kg m^-2^ tephra load is too small to map

Tephra ground loads with 10 and 50% probability of exceedance are shown in Fig. 6. For a 90% probability of exceedance, the area is too small to map. Figure 7 maps the probability of tephra ground loads exceeding the thresholds shown in Table 3 (0.1 kg m^−‍2^, 1 kg m^−2^ and 10 kg m^−2^; the area for 100 kg m^−2^ tephra load is too small to represent). For loads with a 10% probability of exceedance, the hazard footprint ranges from 820 km^2^ for a tephra load of 10 kg m^−2^ to 56,010 km^2^ for a load of 0.1 kg m^−2^ (Table 4).

**Table 4:** Total area impacted at tephra load thresholds (from Table 3) for 10%, 50% and 90% probability of exceedance for modelled VEI 3 and VEI 4 eruptions from Mt. Garibaldi (*Nch’kay’).* A ‘-‘ symbol indicates area is too small to be mapped

Tephra load (kg m^− 2^)	Total area impacted (km^2^)				
	VEI 3		VEI 4		
	10%	50%	10%	50%	90%
0.1	56,010	810	572,820	9360	230
1	8190	250	79,840	1670	100
10	820	-	11,470	450	20
100	-	-	1160	-	-

### Mt. Garibaldi VEI 4 scenario

**Fig. 8:**
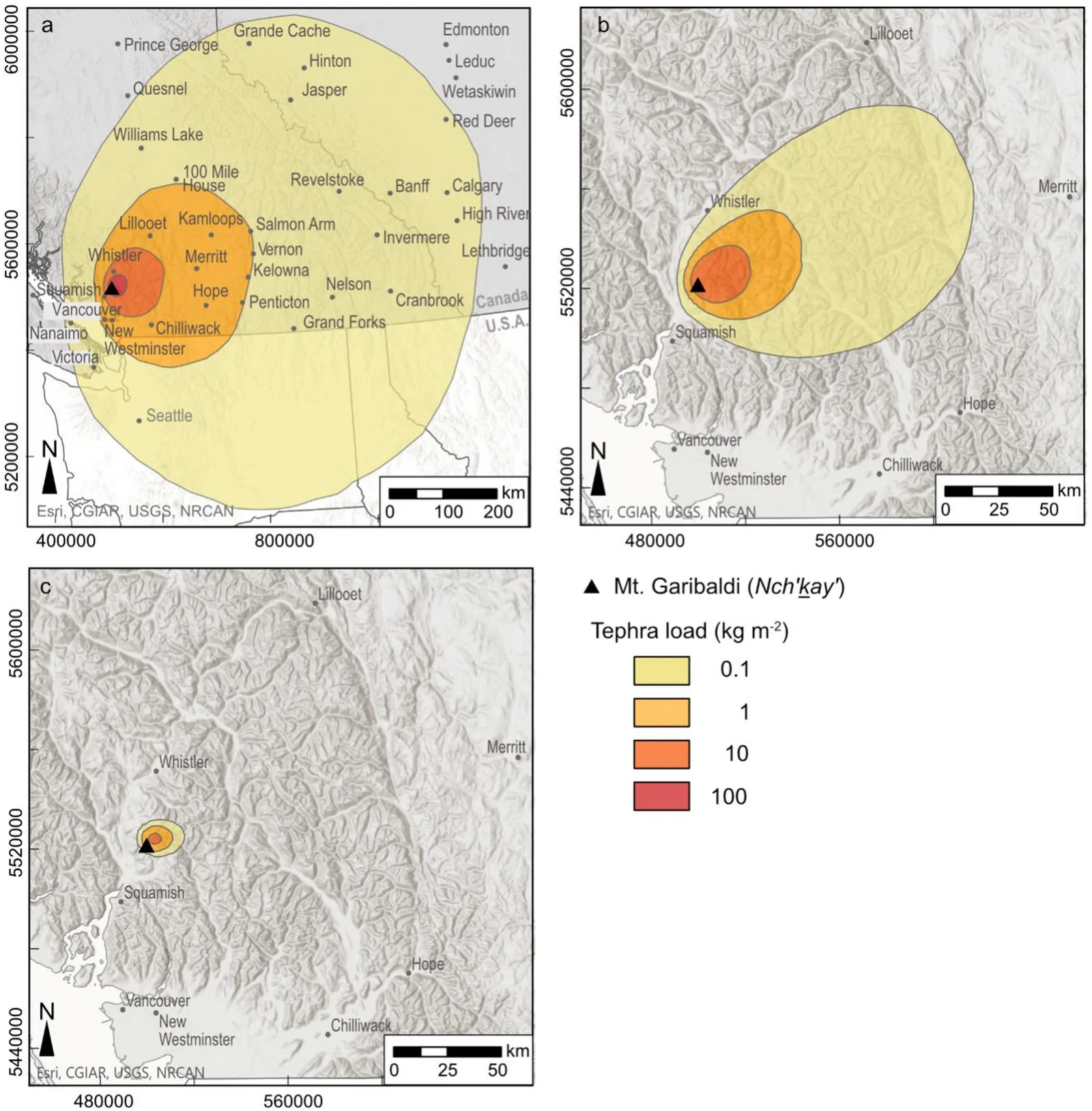
Tephra ground loads from a modelled VEI 4 eruption of Mt. Garibaldi (*Nch’kay’)* with probability of exceedance of **(a)** 10%, **(b)** 50% and **(c)** 90%. Coordinate system UTM 10 N, NAD83 Datum. Note: different scales for each map

**Fig. 9:**
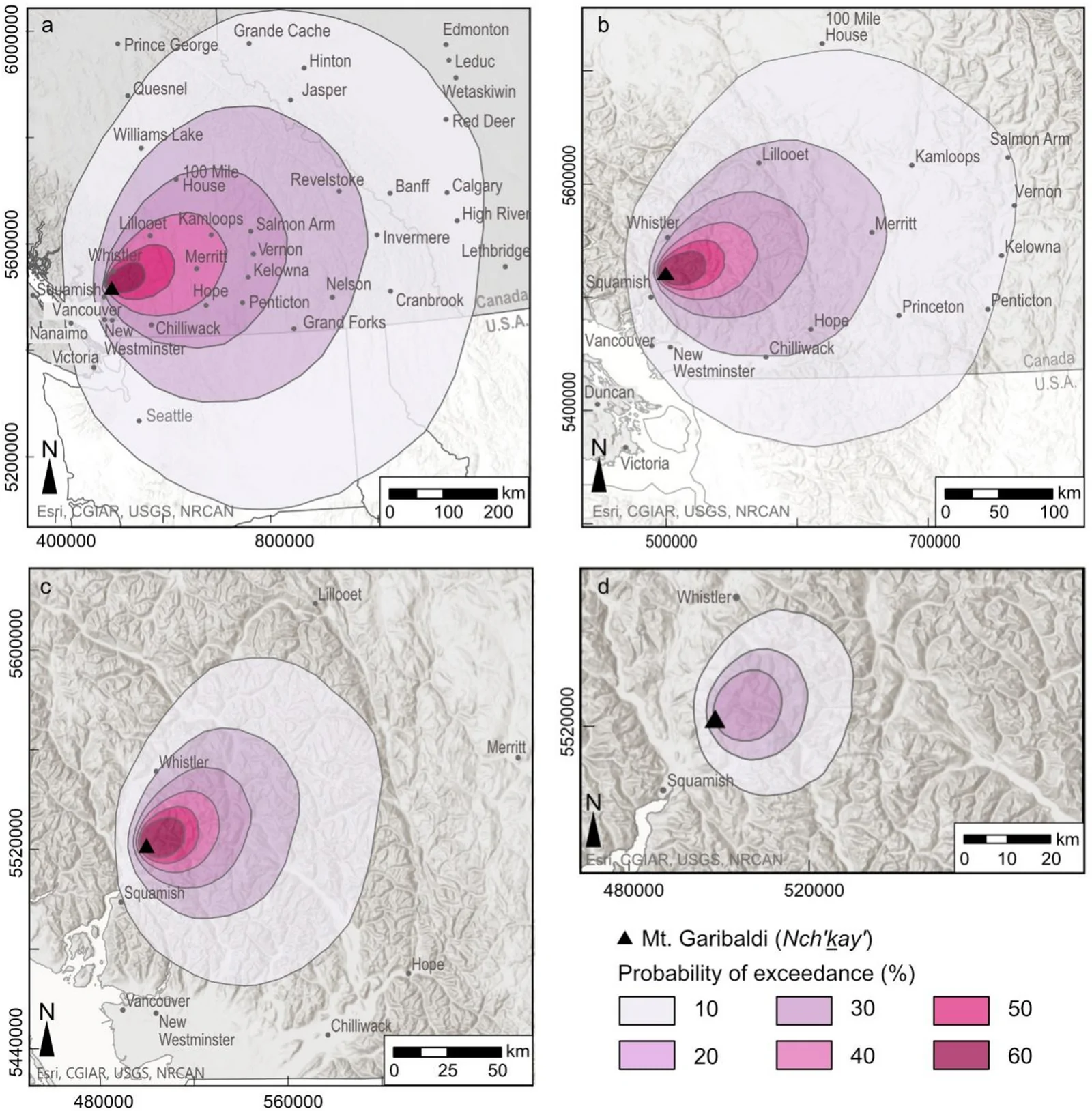
Probability of tephra ground loads from a modelled VEI 4 eruption of Mt. Garibaldi (*Nch’kay’)* exceeding thresholds of **(a)** 0.1 kg m^-2^, **(b)** 1 kg m^-2^, **(c)** 10 kg m^-2^ and **(d)** 100 kg m^-2^. Coordinate system UTM 10 N, NAD83 Datum. Note: different scales for each map

Tephra ground loads with 10 and 50% probability of exceedance are shown in Figs. 8 and 9 maps the probability of tephra ground loads exceeding thresholds of 0.1 kg m^− 2^, 1 kg m^− 2^, 10 kg m^− 2^ and 100 kg m^− 2^. The areas impacted are shown in Table 4 and hazard footprints range from 1160 km^2^ for a tephra load of 100 kg m^− 2^ to 572,820 km^2^ for a load of 0.1 kg m^− 2^ (with a 10% probability of exceedance). We have combined these results into a single one-page hazard map and information brief (Supplementary Material SM4 in Additional File 2).

## Discussion

**Fig. 10:**
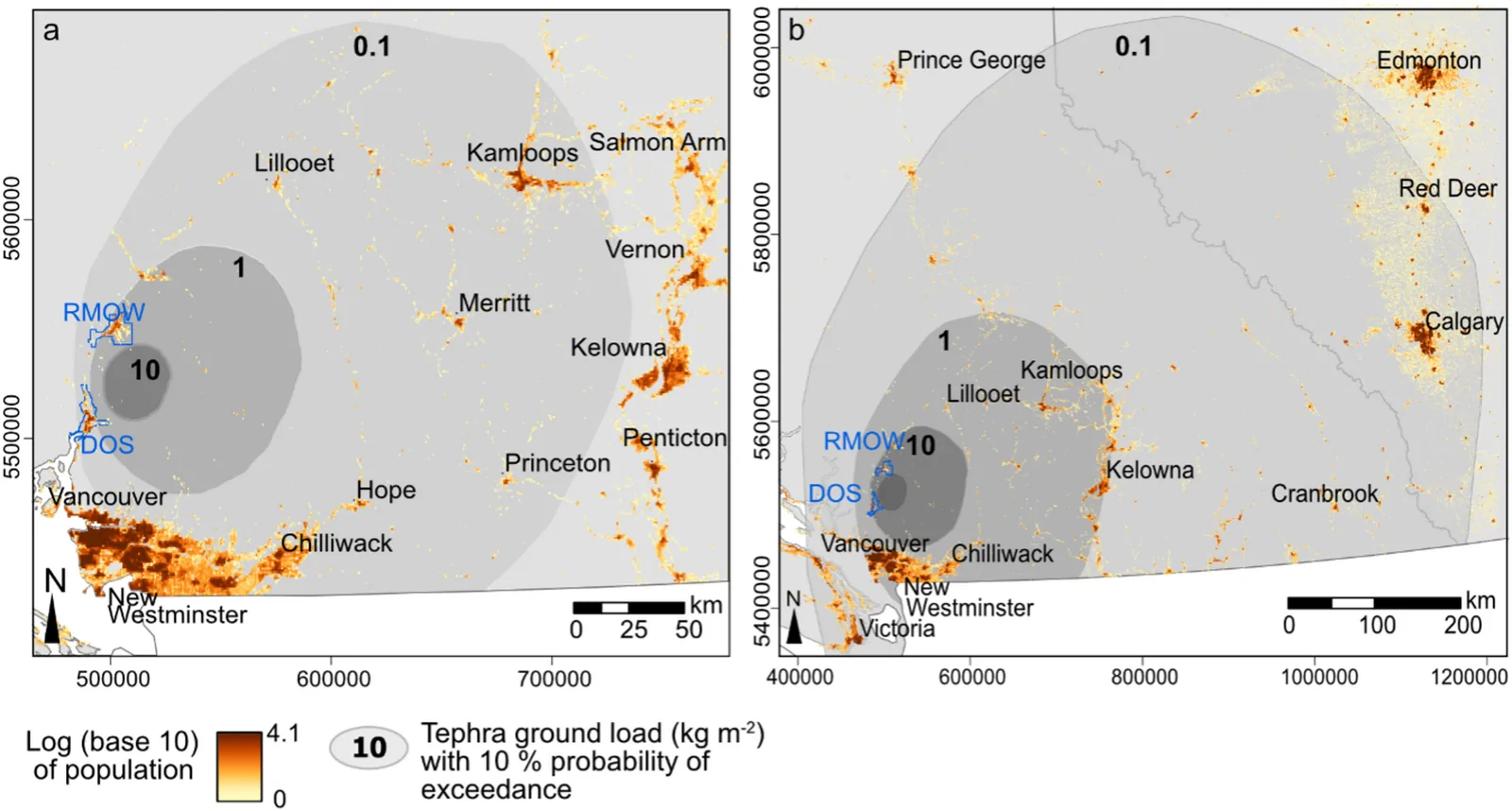
Population in Canada (2024 data from Lebakula et al. 2024) living in areas with 10% probability of exceeding tephra fall ground load thresholds of 0.1, 1, 10 and 100 kg m^-2^, for modelled eruptions of Mt. Garibaldi (*Nch’kay’)*, **(a)** VEI 3, **(b)** VEI 4. DOS = District of Squamish, RMOW = Resort Municipality of Whistler. Coordinate system UTM 10 N, NAD83 Datum. Note: different scales for each map

Our tephra ground accumulation hazard map is the first probabilistic volcanic hazard map using well-constrained source parameters for a Canadian volcano. A full assessment of potential impacts is beyond the scope of this paper and in this discussion we focus only on the number of people who may be affected within Canada. Figure 10 shows the 2024 distribution of population in Canada within the hazard zone (Lebakula et al. 2024). A VEI 4 eruption could potentially impact almost 6.6 million people who live in areas with ≥ 10% probability of exceeding 0.1 kg m^− 2^ tephra ground loading. Furthermore, exposure to the tephra fall hazard is increasing, as within the same region, the population has increased by ~ 30% over the preceding 20 years (from 5.1 million in 2004; Bright et al. 2005). For a VEI 3 eruption, ~ 1.7 million people were at risk in 2024 was compared to ~ 1.3 million in 2004 (Bright et al. 2005; Lebakula et al. 2024). Table 5 shows the number of people living in areas with ≥ 10% probability of exceeding the tephra loads shown in Table 3 (i.e. 0.1, 1, 10, and 100 kg m^− 2^). The overall number of people living in areas at risk of the highest tephra loads (i.e. 10 kg m^− 2^ for the VEI 3 scenario and 100 kg m^− 2^ for the VEI 4 scenario) extending ~ 30 km downwind of the volcano and thus covering the most proximal regions, decreased between 2004 and 2024 (Table 5). However, census data (Statistics Canada 2002, 2023) show populations in the Resort Municipality of Whistler and the District of Squamish (RMOW and DOS shown in Fig. 10) increased by 57 and 67% respectively between 2001 and 2021 (from 8896 to 13982 for RMOW and from 14247 to 23819 for DOS).

**Table 5:** Estimates of 2004 and 2024 population in Canada at risk of tephra ground loads shown in Table 3 (with 10% probability of exceedance) for VEI 3 and VEI 4 eruptions of Mt. Garibaldi (*Nch’kay’*) (Bright et al. 2005; Lebakula et al. 2024)

Tephra ground load (kg m^− 2^)	VEI 3		VEI 4	
	2004 population	2024 population	2004 population	2024 population
0.1	1,282,842	1,669,348	5,131,588	6,570,012
1	15,004	16,779	2,701,700	3,373,094
10	268	14	30,731	40,755
100	0	0	528	14

Tephra fall is, of course, just one hazard from an explosive volcanic eruption and future work must address gravity-driven flows, including lahars and PDCs, which will be impacted by local topography. We have not considered ballistics which typically cause damage and injuries within ~ 5 km from the source (Fitzgerald et al. 2017), as the area proximal to the volcano is very sparsely populated. We have also not considered airborne tephra hazard, e.g. concentrations in the volcanic plume, where ash may remain in the atmosphere for many days. Potential hazard for aircraft is guided by the Volcanic Ash Advisory Centres (e.g. Titos et al. 2022; Pardini et al. 2023; Beckett et al. 2024), which forecast longer-range dispersion, including locations and concentrations of volcanic ash in the atmosphere.

## Conclusion

We have produced the first probabilistic tephra ground accumulation hazard map for VEI 3 and VEI4 eruptions from Mt. Garibaldi. The population living in areas potentially at risk of tephra fall within Canada is increasing, highlighting the importance of understanding tephra fall hazard and assessing exposure and potential impacts. The next steps for this work, in conjunction with the relevant authorities, will address exposure of buildings and other infrastructure to enable appropriate mitigation measures to be considered.

## Supplementary Information

Below is the link to the electronic supplementary material.

Supplementary Material 1

Supplementary Material 2

## Data Availability

All data supporting the findings of this study are available within the paper and its Supplementary Information (Additional Files 1 and 2).
