# Supplementary material for: Tephra fall probabilistic hazard maps for VEI 3 and VEI 4 eruptions at Mt. Garibaldi (Nch’kay’) volcano, British Columbia, Canada
Source: J Appl Volcanol. 2026 Sep 17;15(1):14. doi: 10.1186/s13617-026-00173-8 (PMC13582183; doi:10.1186/s13617-026-00173-8)
Supplement: Supplementary file 1 [file 13617_2026_173_MOESM1_ESM.pdf]

Supplementary Materials for:

Tephra fall probabilistic hazard maps for VEI 3 and VEI 4 eruptions at Mount Garibaldi (*Nch'k̲ay'*) volcano, British Columbia, Canada

Sara Osman[1], Thomas J. Jones[1], Yannick Le Moigne[2,3], Melanie Kelman[2]

[1]Lancaster Environment Centre, Lancaster University, Lancaster, United Kingdom

[2]Natural Resources Canada, Geological Survey of Canada, Vancouver, British Columbia, Canada

[3]Department of Earth Sciences, Simon Fraser University, Burnaby, BC, Canada

*Corresponding author:

Email: s.j.osman@lancaster.ac.uk

Contents of this file:

SM1. Model calibration 2

SM2. Estimation of the magma viscosity 9

SM3. Input parameters sampled for model runs 20

SM1. Model calibration

For all calibration runs we used the Tephra2 dispersion model (Bonadonna et al. 2005; Connor and Connor 2006) with the TephraProb Matlab package (Biass et al. 2016).

Calibration for a VEI 3 eruption

Eruption source parameters used for the VEI 3 calibration are shown in Table S1. We used National Centers for Environmental Prediction/ Department of Energy (NCEP/DOE) Reanalysis II wind data (Kanamitsu et al. 2002). The wind profile for the time of the Mt. Spurr eruption (17th September 1992 08:00 UTC) did not produce a viable model plume (i.e. one where the combination of wind velocity, mass eruption rate and eruption duration resulted in total erupted mass within the specified range). We therefore randomly selected 6-hourly wind profiles from a ten-year dataset (1988 – 1997), where wind direction was consistent with the dispersal direction recorded by the deposit locations (065 – 085 °).

We used the isomass map from McGimsey (2001) as the basis for our calibration, but we did not try to reproduce secondary thickening measured ~ 180 – 350 km downwind. Secondary thickening can be due to aggregation of the finest particles (e.g. Wallace et al. 2013; Tsuji et al. 2020), a bimodal grain size distribution (e.g. Eychenne et al. 2012, 2017), topographic effects on the wind field (e.g. Watt et al. 2015; Eychenne et al. 2017) or contribution from PDC-derived elutriated ash (e.g. Eychenne et al. 2015; Brown et al. 2022), the distinction between which is beyond the scope of the physics captured by the Tephra2 model. Best-fit model parameters selected for our VEI 3 simulations were:

- Plume model (β distribution) parameters: α = 10, β = 2.
- Diffusion coefficient: 7500 m^2 s^{-1}.
- Fall time threshold: 9700 s (Figure S1 a-c).

Calibration for a VEI 4 eruption

Eruption source parameters used for the VEI 4 calibration are shown in Table S2. We used NCEP/National Center for Atmospheric Research (NCEP/NCAR) Reanalysis I wind data (Kalnay et al. 1996), which provides wind velocity data at 17 atmospheric pressure levels. Again, there was no viable modelled plume using the wind profile for the specific time of the Mount St Helens eruption (18th May 1980 15:30 UTC). We therefore randomly selected 6-hourly wind profiles from the 1976 – 1985 dataset where wind direction was 60 – 80 °, consistent with deposit locations. The Reanalysis II data used for the Mt. Spurr calibration is only available from January 1979 (when modern satellite data ingestion started) and so for this calibration we used Reanalysis I, which is available from 1948, but uses different pre- and post-1979 data assimilation patterns.

We did not try to completely match observed ground tephra loading. Tephra2 can only ingest unimodal grain size distributions (GSDs) but the Mount St Helens deposit had a bimodal GSD (Carey and Sigurdsson 1982). In addition, secondary thickening has been interpreted as fallout from a co-PDC plume (Eychenne et al. 2015), while we only modelled fallout from the main plume. We calibrated our model using the isomass map from Sarna-Wojcicki (1981), taking into account that the mass deposited from plume fallout 150 – 320 km from source is estimated at only ~ 50 % of the total measured deposit (Eychenne et al. 2015). Best-fit model parameters selected for our VEI 4 simulations were:

- Plume model (β distribution) parameters: $\alpha = 3$, $\beta = 2$.
- Diffusion coefficient: 7500 $m^2\ s^{-1}$.
- Fall time threshold: 9700 s (Figure S1 d-f).

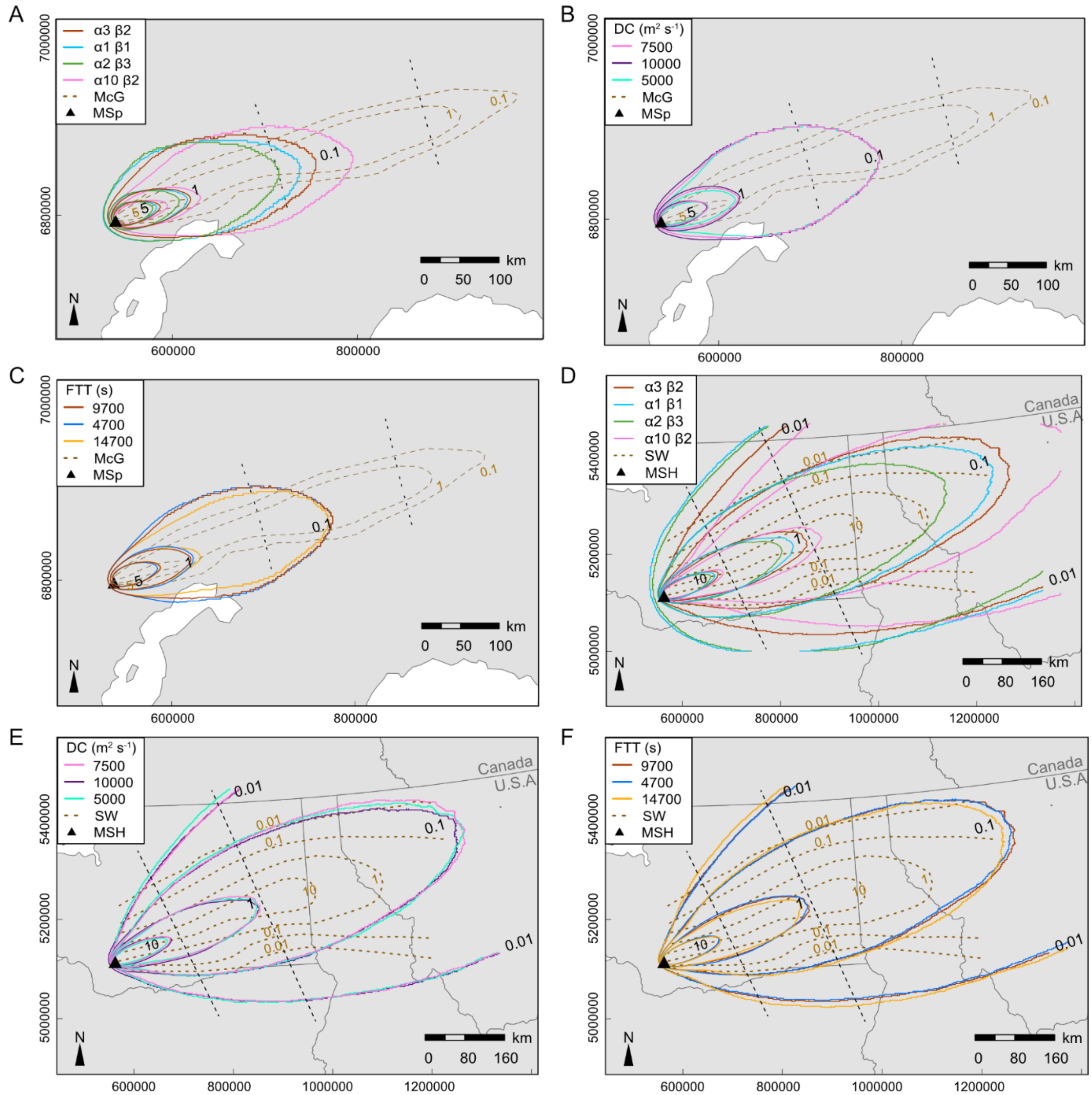

Figure S1 Calibration runs showing test results and published isomass data. A-C) Mt. Spurr September 1992 VEI 3 calibration, D-F) Mount St Helens 1980 VEI 4 calibration. A and D) plume model, B and E) diffusion coefficient (DC), C and F) fall-time threshold (FTT). All isomass contours are in kg m^{-2}. The regions between the dashed black lines indicate areas of secondary thickening (Eychenne et al. 2015, 2017) McG = McGimsey et al. 2001; SW = Sarna-Wojcicki et al. 1981; MSp = Mt. Spurr volcano; MSH = Mount St Helens volcano

Table S1 Input parameters for model calibration for VEI 3 event, based on Mt. Spurr 17th September 1992 eruption

Parameter	Values	Comments
**Volcano**		
Vent easting	540146.46	UTM 5N
Vent northing	6795676.23	
Vent height (m a.s.l.)	3374	
**Computational grid**		
Resolution (m)	2000	
Mean elevation (m a.s.l.)	1000	
**Source parameters**		
Plume height (km a.s.l.)	13 – 15<br>Sampling: uniform	14 km (Rose et al. 1995)
Erupted mass (kg)	3.5–4.5 x 10^{10}	Vol. 56 x10^6 m^3 and 700 kg m^{-3} (Neal et al. 1995). Secondary thickening ~ 180–350 km downwind (Eychenne et al. 2017)
Eruption duration (h)	2–4	3 h (Rose et al. 1995); 3.6 h (Eichelberger et al. 1995)
**Wind profiles**		
Wind dataset	01-Jan-1988 – 31 Dec 1997	(Kanamitsu et al. 2002). NCEP/DOE Reanalysis II data provided by NOAA PSL(https://psl.noaa.gov)
Total number of wind profiles	14612	4 wind profiles per day
Wind direction (Min/max) (° clockwise from N)	65–85	Range tested 55–100 °
Troposphere height (km a.s.l.)	12.3	11.8 – 12.8 (Durant and Rose 2009)
**Grain size and density**		
Max and min (Φ)	-5 10	
Median (Φ)	3.0–4.0	(Durant and Rose 2009)
Standard deviation (Φ)	1.5–2.5	
Aggregation coefficient (0 – 1)	0.2 – 0.8	Aggregation of particles ≤ 5 Φ
Lithic density (kg m^{-3})	2500	Typical values: 2300–2700 (Bonadonna and Phillips 2003)
Pumice density (kg m^{-3})	1750	1530 – 2010 kg m^{-3} (Neal et al. 1995)
**Model constants**		
Eddy constant (m^2 s^{-1})	0.04	(Connor et al. 2011)
Column integration steps	100	
Particle integration steps	50	
Number of runs	1000	
**Model parameters tested during calibration**		**Best-fit values selected**
α, β parameters of the β distribution	1, 1 3, 2<br>2, 3 10, 2	10, 2
Diffusion coefficient (m^2 s^{-1})	5000–10000	7500
Fall time threshold (s)	4700–14700	9700

Table S2 Input parameters for model calibration for VEI 4 event, based on Mount St Helens 18th May 1980 eruption

Parameter	Values	Comments
**Volcano**		
Vent easting	562069.98	UTM 10N
Vent northing	5115626.70	
Vent height (m a.s.l.)	2500	(Werner Kieffer 1981)
**Computational grid**		
Resolution (m)	2000	
Mean elevation (m a.s.l.)	1000	
**Source parameters**		
Plume height (km a.s.l.)	10–24<br>Sampling: uniform	10–14 km (Mastin et al. 2023); 14–24 km (Carey and Sigurdsson 1982).
Erupted mass (kg)	$10^{11} – 10^{12}$	4.9 x10^{11} – 5.5 x 10^{11} kg (Sarna-Wojcicki et al. 1981). Eychenne et al. (2015) estimate 6.2 x 10^{10} kg from co-PDC plume in zone 150 – 320 km from source.
Eruption duration (h)	8–10	Approx 9h (Harris et al. 1981)
**Wind profiles**		
Wind dataset	01-Jan-1976 – 31 Dec 1985	(Kalnay et al. 1996). NCEP/NCAR Reanalysis I data provided by NOAA PSL(https://psl.noaa.gov)
Total number of wind profiles	14612	4 wind profiles per day
Wind direction (Min/max) (° clockwise from N)	60–80	Range tested 15 – 135 °
Troposphere height (km a.s.l.)	10.5	(Liu et al. 2014)
**Grain size and density**		
Max and min (Φ)	-5 9	
Median (Φ)	2.5–4.5	(Carey and Sigurdsson 1982)
Standard deviation (Φ)	1.4–3.4	
Aggregation coefficient (0 – 1)	0.2–0.8	Aggregation of particles ≤ 5 Φ
Lithic density (kg m^{-3})	2500	2300–2700 (Bonadonna and Phillips 2003)
Pumice density (kg m^{-3})	1500	Estimated (Bonadonna and Phillips 2003)
**Model constants**		
Eddy constant ($m^2 s^{-1}$)	0.04	(Connor et al. 2011)
Column integration steps	100	
Particle integration steps	50	
Number of runs	1000	
**Model parameters tested during calibration**		**Best-fit values selected**
α, β parameters of the β distribution	1, 1 3, 2<br>2, 3 10, 2	3, 2
Diffusion coefficient ($m^2 s^{-1}$)	5000–10000	7500
Fall time threshold (s)	4700–14700	9700

References

Biass S, Bonadonna C, Connor LJ, Connor CB (2016) TephraProb: a Matlab package for probabilistic hazard assessments of tephra fallout. J Appl Volcanol 5:10. https://doi.org/10.1186/s13617-016-0050-5

Bonadonna C, Connor CB, Houghton BF, et al (2005) Probabilistic modeling of tephra dispersal: hazard assessment of a multiphase rhyolitic eruption at Tarawera, New Zealand. J Geophys Res Solid Earth 110:B03203. https://doi.org/10.1029/2003JB002896

Bonadonna C, Phillips JC (2003) Sedimentation from strong volcanic plumes. J Geophys Res Solid Earth 108. https://doi.org/10.1029/2002JB002034

Brown RJ, Hernández W, Escobar D, et al (2022) Reconstruction of the 29th December 2013 eruption of San Miguel volcano, El Salvador, using video, photographs, and pyroclastic deposits. Volcanica 5:271–293. https://doi.org/10.30909/vol.05.02.271293

Carey SN, Sigurdsson H (1982) Influence of particle aggregation on deposition of distal tephra from the May 18, 1980, eruption of Mount St. Helens volcano. J Geophys Res Solid Earth 87:7061–7072. https://doi.org/10.1029/JB087iB08p07061

Connor L, Connor C, Courtland L (2011) Tephra2 users manual. University of South Florida, Tampa, FL

Connor LJ, Connor CB (2006) Inversion is the key to dispersion: understanding eruption dynamics by inverting tephra fallout. In: Mader H, Coles; SG, Connor; CB, Connor LJ (eds) Statistics in Volcanology. GSL, London, pp 231–242

Durant AJ, Rose WI (2009) Sedimentological constraints on hydrometeor-enhanced particle deposition: 1992 Eruptions of Crater Peak, Alaska. J Volcanol Geotherm Res 186:40–59. https://doi.org/10.1016/j.jvolgeores.2009.02.004

Eichelberger JC, Keith TEC, Miller TP, Nye CJ (1995) The 1992 eruptions of Crater Peak Vent, Mount Spurr Volcano, Alaska: chronology and summary. In: Keith TEC (ed) The 1992 eruptions of Crater Peak vent, Mount Spurr Volcano, Alaska. USGS, pp 1–18

Eychenne J, Cashman K, Rust A, Durant A (2015) Impact of the lateral blast on the spatial pattern and grain size characteristics of the 18 May 1980 Mount St. Helens fallout deposit. J Geophys Res Solid Earth 120:6018–6038. https://doi.org/10.1002/2015JB012116

Eychenne J, Le Pennec J-L, Troncoso L, et al (2012) Causes and consequences of bimodal grain-size distribution of tephra fall deposited during the August 2006 Tungurahua eruption (Ecuador). Bull Volcanol 74:187–205. https://doi.org/10.1007/s00445-011-0517-5

Eychenne J, Rust AC, Cashman K V., Wobrock W (2017) Distal enhanced sedimentation from volcanic plumes: insights from the secondary mass maxima in the 1992 Mount Spurr fallout deposits. J Geophys Res Solid Earth 122:7679–7697. https://doi.org/10.1002/2017JB014412

Harris DH, Rose WI, Roe R, Thompson MR (1981) Radar observations of ash eruptions. In: Lipman PW, Mullineaux DR (eds) The 1980 eruptions of Mount St. Helens, Washington. USGS, Washington DC, pp 323–333

Kalnay E, Kanamitsu M, Kistler R, et al (1996) The NCEP/NCAR 40-year reanalysis project. Bull Am Meteorol Soc 77:437–471. https://doi.org/10.1175/1520-0477(1996)077<0437:TNYRP>2.0.CO;2

Kanamitsu M, Ebisuzaki W, Woollen J, et al (2002) NCEP–DOE AMIP-II Reanalysis (R-2). Bull Am Meteorol Soc 83:1631–1644. https://doi.org/10.1175/BAMS-83-11-1631

Liu Y, Xu T, Liu J (2014) Characteristics of the seasonal variation of the global tropopause revealed by COSMIC/GPS data. Adv Sp Res 54:2274–2285. https://doi.org/10.1016/j.asr.2014.08.020

Mastin LG, Carey SN, Van Eaton AR, et al (2023) Understanding and modeling tephra transport: lessons learned from the 18 May 1980 eruption of Mount St. Helens. Bull Volcanol 85:4. https://doi.org/10.1007/S00445-022-01613-0

McGimsey RG, Neal CA, Riley CM (2001) Areal distribution, thickness, mass, volume, and grain size of tephra-fall deposits from the 1992 eruptions of Crater Peak Vent, Mt. Spurr Volcano, Alaska. U.S. Geological Survey Open-File Report 01-370. USGS, Anchorage

Neal CA, McGimsey RG, Gardner CA, et al (1995) Tephra-fall deposits from the 1992 eruptions of Crater Peak, Mount Spurr Volcano, Alaska: a preliminary report on distribution, stratigraphy, and composition. In: Keith TEC (ed) The 1992 eruptions of Crater Peak vent, Mount Spurr Volcano, Alaska. USGS, pp 65–79

Rose WI, Kostinski AB, Kelley L (1995) Real-time C-band radar observations of 1992 eruption clouds from Crater Peak, Mount Spurr volcano, Alaska. In: Keith TEC (ed) The 1992 eruptions of Crater Peak vent, Mount Spurr Volcano, Alaska. USGS, pp 19–26

Sarna-Wojcicki AM, Shipley S, Waitt RB, et al (1981) Areal distribution, thickness, mass, volume and grain size of air-fall ash from the six major eruptions of 1980. In: Lipman PW, Mullineaux DR (eds) The 1980 Eruptions of Mount St. Helens. USGS, Washington DC, pp 577–600

Tsuji T, Nishizaka N, Ohnishi K (2020) Influence of particle aggregation on the tephra dispersal and sedimentation from the October 8, 2016, eruption of Aso volcano. Earth, Planets Sp 72:104. https://doi.org/10.1186/s40623-020-01233-y

Wallace KL, Schaefer JR, Coombs ML (2013) Character, mass, distribution, and origin of tephra-fall deposits from the 2009 eruption of Redoubt Volcano, Alaska—highlighting the significance of particle aggregation. J Volcanol Geotherm Res 259:145–169. https://doi.org/10.1016/j.jvolgeores.2012.09.015

Watt SFL, Gilbert JS, Folch A, et al (2015) An example of enhanced tephra deposition driven by topographically induced atmospheric turbulence. Bull Volcanol 77:35. https://doi.org/10.1007/s00445-015-0927-x

Werner Kieffer S (1981) Fluid dynamics of the May 18 blast at Mount St. Helens. In: Lipman PW, Mullineaux DR (eds) The 1980 eruptions of Mount St. Helens, Washington. USGS, Washington DC, pp 379–400

SM2. Estimation of the magma viscosity

The composition of Alice Ridge block-and-ash flow deposit (Figure S2) has been chosen as a representative dacitic composition for Mount Garibaldi. This composition is used for the determination of the grain size distribution needed for the Tephra2 simulations.

Brief Description of the Alice Ridge Block-and-Ash Deposit

The Alice Ridge block-and-ash is a voluminous (~1 km^3) pinkish-grey poorly sorted, bedded, matrix supported block-and-ash flow deposit located on the southern flank of Atwell Peak (Figure S2A, B). This deposit was emplaced during numerous dome syn-eruptive collapse events at 39.0 ± 2.8 ka. The blocks consist of light to medium grey, sparsely vesiculated, breadcrusted, and radially jointed plagioclase-amphibole-orthopyroxene-phyric dacite up to ~20 m across (Figure S2C, D).

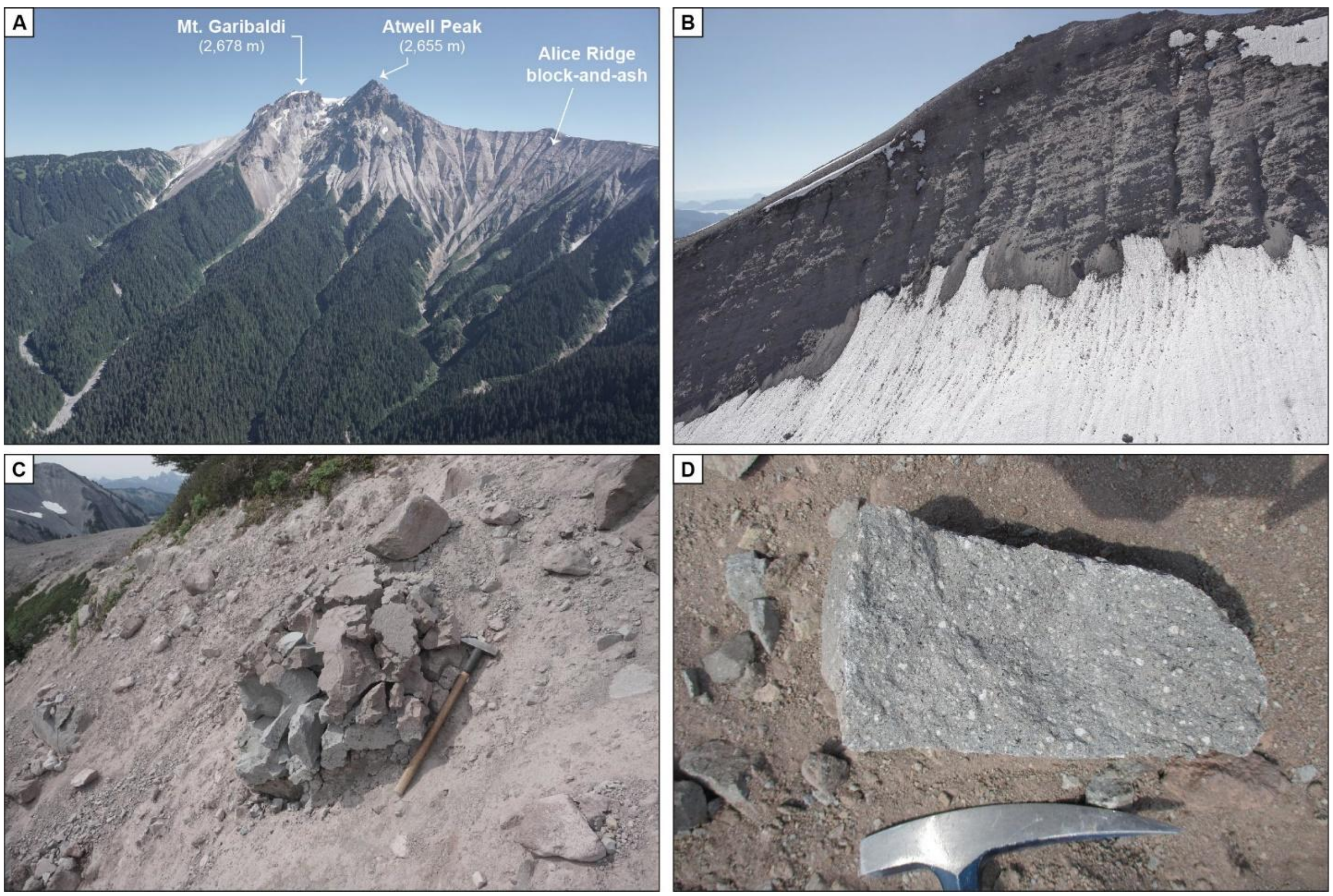

Figure S2 A: View of Mount Garibaldi and the upper Alice Ridge (right side), looking east. The Alice Ridge block-and-ash flow deposit is located on the southern flank of Atwell Peak and is approximately 500-700 m thick. **B**: Close up view of the Alice Ridge block-and-ash. Note the pronounced bedding. Blocks are up to 15 m in diameter on this photo. **C**: A breadcrusted and radially jointed dacite block. **D**: Fresh looking interior of a dacite block sampled for whole rock geochemistry and thin section.

To estimate the grain sizedistribution, we first need to constrain the magma apparent viscosity. This was done in several steps:

1. We estimated the median bulk rock composition of the Alice Ridge block-and-ash flow deposit.
2. We measured the proportion of each phenocryst phase and their composition.
3. We calculated the melt composition accounting for the removal of the phenocryst phases and their respective proportions.
4. The magma eruption temperature and water content was estimated from amphibole-only thermometer and hygrometer.
5. The final magma viscosity was estimated by integrating the effect of the suspended phenocrysts to the melt viscosity.

Below, we detail the methodology and show the intermediate results.

Analytical Methods

14 samples were collected for whole rock geochemistry. These samples were carefully selected from the less altered, non-weathered and less vesiculated radially jointed dacite blocks. Major element contents of 14 samples were analysed by ActLabs (Kamloops, BC) using lithium borate fusion ICP-MS with Fe_2O_3(T) measured as total iron. Ferrous iron (FeO) was measured separately by fluorine titration. Three thin sections from the interior of blocks were confectioned by Vancouver Petrographic for petrographic investigations. The proportions of phenocrysts, vesicles, and groundmass (microlites and interstitial glass) was determined by manually tracing the crystals on Adobe Illustrator. The proportions, in vol.%, of each phenocryst phase, groundmass, and vesicles were estimated using ImageJ (Schneider et al., 2012). Only the largest vesicles that were clearly visible under the petrographic microscope at the ×2.5 and ×5 objectives were considered.

Crystal compositions were analysed using a JEOL JXA 8530FPlus field emission electron microprobe, equipped with 5 tuneable wavelength dispersive spectrometers and 2 SDD energy dispersive spectrometers, housed in the Electron Microbeam and X-Ray Diffraction Facility (EMXDF) of the Department of Earth, Ocean and Atmospheric Sciences, The University of British Columbia (Vancouver, British Columbia, Canada). The following phases were analysed: plagioclase (n = 22), amphibole (n = 36), orthopyroxene (n = 27), and oxide (n = 8). We analysed Si, Ti, Al, Fe, Mn, Mg, Ca, Na, K, Cr, Ni, F and Cl in amphibole with an accelerating voltage of 15 kV, a beam current of 15 nA, and a beam diameter of 5 μm. Peak counting times were 10 seconds for Al and Ca; 20 seconds for Si, F, Na, and K; 30 seconds for Cl, Mg, and Ti; and 40 seconds for Fe and Mn. For the orthopyroxene, the analytical conditions consisted

of an accelerating voltage of 15 kV, a beam current of 20 nA, and a beam diameter of 2 µm. Peak counting times were 20 seconds for all elements. For Fe-Ti oxide, an accelerating voltage of 15 kV, a beam current of 20 nA, and a beam diameter of 1 µm were used. Peak counting times were 10 seconds for Si and Ca, 20 seconds for Cr, Al, Ti, Mg, Fe, Mn, and Ni. For plagioclase, the analytical conditions used were an accelerating voltage of 15 kV, a beam current of 20 Na, and a beam diameter of 2 µm.

Whole Rock Geochemistry and Petrology

The 14 whole rock analyses were normalized to 100 wt.% on a LOI-free basis (Le Bas et al., 1986; Table S3). The Alice Ridge block-and-ash is calk-alkaline dacitic in composition with a narrow compositional range (SiO_2 = 64.3-66.6 wt.%, MgO = 2.0-2.1 wt.%; Figure S3). We used the median composition for the calculation of the calculation of the magma viscosity.

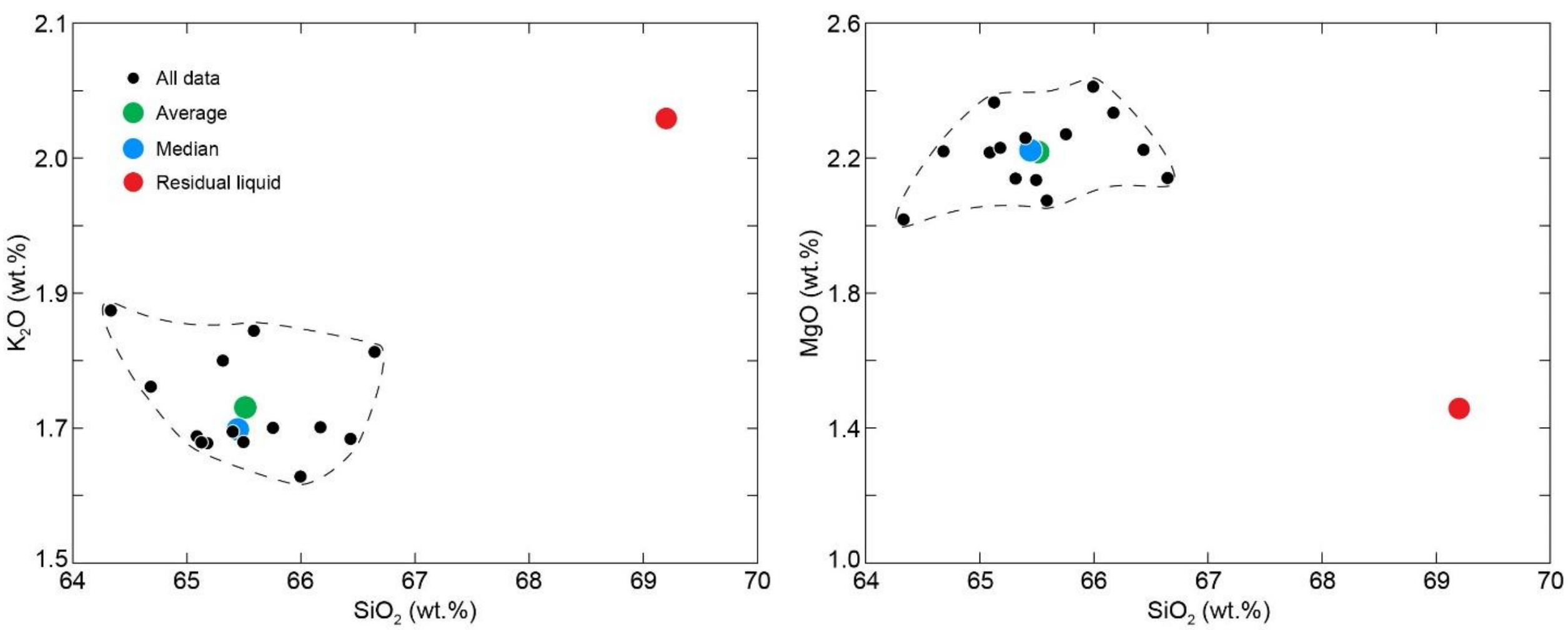

Figure S3 Two binary diagrams showing the whole rock compositions of the Alice Ridge block-and-ash dacite. The red circle corresponds to the recalculated residual liquid composition that is used to calculate the magma viscosity needed for the Tephra2 simulations. See methods in the text.

The Alice Ridge block-and-ash dacite comprises phenocrysts of plagioclase, amphibole, and orthopyroxene. The compositions and proportions of these phenocryst phases are reported in Table S4 and S5, respectively. The variation in plagioclase proportion can be explained by a few large, isolated phenocrysts (Figure S4).

Table S3 Whole rock compositions of the Alice Ridge block-and-ash flow deposit. The median composition is used for calculation of the magma viscosity needed for determination of the grain size distribution. X and Y are the sample locations reported in metres (coordinate system: UTM Zone 9N; WGS 84 datum). LOI = loss on ignition

Sample ID	X (m)	Y (m)	SiO_2	TiO_2	Al_2O_3	FeO_T	MnO	MgO	CaO	Na_2O	K_2O	P_2O_5	Total	LOI	Original Total
HHB07-04-02	499406	5520009	65.50	0.49	16.73	4.24	0.09	2.13	4.72	4.23	1.68	0.18	100	1.07	100.60
22AW-010	499787	5518987	64.68	0.52	16.66	4.61	0.09	2.22	4.74	4.54	1.76	0.17	100	1.63	100.20
22AW-011	499851	5518945	65.32	0.49	16.64	4.68	0.08	2.14	4.46	4.20	1.80	0.19	100	1.33	98.00
22AW-011	499851	5518945	64.33	0.46	17.14	5.02	0.09	2.02	4.34	4.54	1.87	0.19	100	1.15	98.50
22AW-013	499643	5518527	65.09	0.50	17.25	3.95	0.08	2.22	4.72	4.33	1.69	0.17	100	0.65	99.25
22AW-013	499643	5518527	65.18	0.50	16.97	4.04	0.08	2.23	4.74	4.40	1.68	0.18	100	0.83	98.80
22AW-016	499831	5518119	65.13	0.49	16.99	4.12	0.09	2.37	4.65	4.32	1.68	0.17	100	1.77	99.67
22AW-017	499139	5518105	65.40	0.51	17.01	3.82	0.08	2.26	4.71	4.33	1.69	0.18	100	2.06	97.98
22KLA-001	499867	5518866	65.76	0.49	16.72	3.90	0.09	2.27	4.66	4.23	1.70	0.19	100	1.30	98.12
22YLM-003	499770	5519017	66.17	0.49	16.20	3.99	0.09	2.33	4.68	4.17	1.70	0.18	100	1.79	98.52
22YLM-005	499522	5519198	66.00	0.47	16.27	4.07	0.09	2.41	4.75	4.14	1.63	0.18	100	1.40	98.64
22YLM-007	498774	5518510	65.59	0.47	16.74	3.95	0.08	2.07	4.59	4.52	1.84	0.15	100	0.51	101.00
22YLM-018	501428	5519834	66.44	0.48	16.14	3.88	0.08	2.22	4.80	4.12	1.68	0.16	100	1.43	97.91
22YLM-045	500090	5520584	66.65	0.47	16.09	3.81	0.09	2.14	4.60	4.15	1.81	0.18	100	1.40	99.26
Average			65.52	0.49	16.68	4.15	0.09	2.22	4.65	4.30	1.73	0.18			
Median			65.45	0.49	16.73	4.01	0.09	2.22	4.69	4.28	1.70	0.18			
STD			0.62	0.02	0.36	0.35	0.00	0.11	0.12	0.14	0.07	0.01			

Table S4 Phenocryst proportions (in vol.%) of three Alice Ridge block-and-ash thin sections. The proportions are normalised to 100 wt.% on a vesicle-free basis. The thin section scans and their respective phenocryst tracing are shown on Figure S4.

Sample ID	Plagioclase	Amphibole	Orthopyroxene	Oxides	Groundmass
22YLM-005	18.6	4.2	2.0	0.3	74.8
22AW-013	14.0	3.8	2.1	0.1	80.0
22AW-016	17.9	3.8	2.0	0.4	75.9

Table S5 Average phenocryst compositions. n refers to the number of analyses. Values in brackets are 1 standard deviation

Phenocryst Phase	SiO_2	TiO_2	Al_2O_3	FeO	MnO	MgO	CaO	Na_2O	K_2O	P_2O_5	Total
Plagioclase	55.86	-	27.11	0.30	-	0.02	10.02	5.76	0.22	-	99.29
(n = 22)	(1.82)	-	(1.22)	(0.06)	-	(0.01)	(1.48)	(0.80)	(0.07)	-	
Amphibole	45.01	1.89	9.75	11.02	0.19	15.36	11.33	2.06	0.39	-	97.00
(n = 36)	(1.56)	(0.26)	(1.69)	(1.31)	(0.07)	(0.75)	(0.24)	(0.29)	(0.15)	-	
Orthopyroxene	53.82	0.12	1.02	16.69	0.66	25.35	1.56	0.03	-	-	99.26
(n = 27)	(0.49)	(0.04)	(0.45)	(3.20)	(0.25)	(2.39)	(3.92)	(0.05)	-	-	
Oxide	0.04	7.04	2.21	82.42	0.42	1.78	0.02	-	-	-	93.93
(n = 8)	(0.02)	(0.46)	(0.25)	(0.78)	(0.03)	(0.17)	(0.04)	-	-	-	

Calculation of the Temperature and Water Contents

The temperature of the magma upon eruption is calculated using the amphibole-only thermometer of Ridolfi and Renzulli (2012) updated by Ridolfi (2021). This thermometer is a multi-element regression model based on the amphibole stoichiometry and has an associated error of ± 22 °C, which represents the principal source of uncertainty on the temperature estimates. This thermometer could be applied for amphibole-bearing calk-alkaline magmas in subduction related settings. The 36 analysed amphibole compositions yield a temperature range of 822 to 920 °C, with a mean of 884 °C and a standard deviation of 36 °C. The corresponding standard error of the mean is ± 6 °C, showing that the average temperature is relatively well constrained by the amphibole dataset. Combining the model calibration uncertainty with the standard error of the mean results in an overall uncertainty of approximately ± 23 °C for the reported calculated average temperature. To account for the observed variability among amphibole compositions and the uncertainty associated with the thermometer calibration, we adopted a conservative temperature range of 820-900 °C in our magma viscosity estimates (see next sections).

The magma water content is estimated using the amphibole-only hygrometer of Ridolfi and Renzulli (2012) updated by Ridolfi (2021). Similarly to the thermometer, this hygrometer is based on a multi-element regression model. The error associated with this hygrometer is ± 14%, which is the dominant source of uncertainty in the calculated dissolved H_2O contents. In our case, the calculated dissolved water content range between ~4.7 and ~6.6 wt.%. These values represent the dissolved water content in the magma at the time of amphibole crystallization.

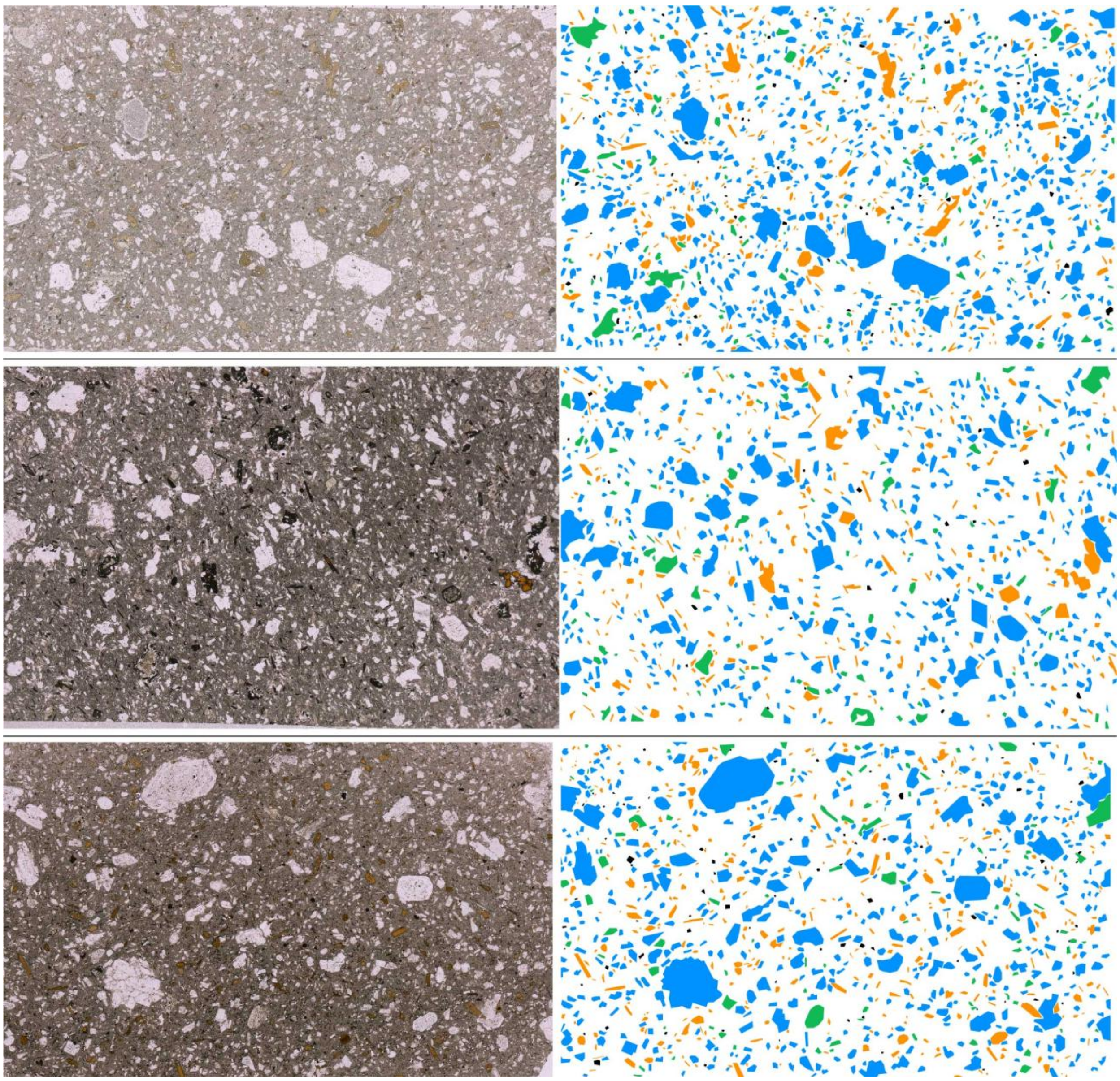

Figure S4 Photomicrograph scans of three representative thin sections of the Alice Ridge block-and-ash flow deposit. On the right side are highlighted the phenocrysts of each thin section. Plagioclase in blue, amphibole in orange, orthopyroxene in green, oxide in black, and groundmass in white. The field of view of each thin section is approximately 23 × 40 mm

Calculation of the Magma Viscosity

The melt viscosity was estimated using the Giordano et al. (2008) model (GRD) which is based on a non-Arrhenian temperature dependence of viscosity and is valid for a large range of silicate melt compositions. The model is expressed in the form of the Vogel-Fulcher-Tamman (VFT) equation (Vogel, 1921; Fulcher, 1925; Tamman and Hess, 1926; Giordano et al., 2008):

$$\log(\eta_{melt}) = A + \frac{B}{T(°K) - C}$$

where the constants A, B, and C are fitting parameters specific to the melt composition (e.g., combinations of major oxides and volatile components in mol.%; Giordano et al., 2008). The composition of the melt was estimated assuming removal of the phenocryst phases from the normalized median whole rock composition relative to their proportions on a vesicle-free basis (see details in Table S6). This calculated melt composition is rhyodacitic in composition with SiO_2 = 69.2 wt.% (Figure S3; Table S3).

Melt viscosities are highly sensitive to the amount of dissolved water within the melt. To apply the GRD model, we used the water content estimated from the amphibole-only hygrometer (4.7 – 6.6 wt.%). Magma loses water through degassing as it ascends, as was shown for the Mount St. Helens 1980 eruption (e.g. Rutherford et al. 1985; Rutherford and Hill 1993; Seligman et al. 2018). The Mount St. Helens dacite lost an estimated ~4 wt.% during ascent, with 0.3 – 2.4 wt.% water measured in groundmass glass compared to 4.6 – 6.4 wt.% in unruptured melt inclusions (Blundy and Cashman, 2005). We therefore reduced the calculated water content by 4 wt.% and used 0.5 – 4.5 wt.% for the melt viscosity calculations. The results of the GRD viscosity model are presented in Table S7.

The crystal effect on the viscosity is directly related to the number of suspended crystals in the magma. There are several methods to model the relative effect of crystals in the melt (e.g., Krieger and Dougherty, 1959; Mueller et al., 2010; Chevrel et al., 2013). Here, we use Equation 2.4 from Mueller et al. (2010) based on Maron and Pierce (1956):

$$\eta_r = \left(1 - \frac{\emptyset}{\emptyset_m}\right)^{-2}$$

where, $\emptyset_m$, refers the maximum packing of spherical particles and considered to be equal to 0.55 (Mader et al.., 2013). In our case, the total proportion of phenocrysts is ~23 vol.%. This corresponds to $\eta_r = 2.97$, where $\eta_{magma} = \eta_{melt} \times \eta_r$.

The final calculated magma viscosities are presented in Table S7. The minimum and maximum apparent magma viscosity used for the grain size distribution calculations are 1.71×10^5 and 1.75×10^8 Pa s, respectively (Table S7).

Table S6 Calculation of the residual melt composition assuming removal of the phenocryst phase from the median whole rock composition

	SiO_2	TiO_2	Al_2O_3	FeO_T	MnO	MgO	CaO	Na_2O	K_2O	P_2O_5	Total
Median whole rock composition[a]	65.45	0.49	16.73	4.01	0.09	2.22	4.69	4.28	1.70	0.18	99.83
Normalized	65.56	0.49	16.76	4.02	0.09	2.23	4.70	4.28	1.70	0.18	100.00
Phenocryst compositions[b]:											
Plagioclase (17.9 vol.%)	55.86	nd	27.11	0.30	nd	0.02	10.02	5.76	0.22	nd	99.29
Amphibole (3.8 vol.%)	45.01	1.89	9.75	11.02	0.19	15.36	11.33	2.06	0.39	nd	97.00
Orthopyroxene (2.0 vol.%)	53.82	0.12	1.02	16.69	0.66	25.35	1.56	0.03	nd	nd	99.26
Oxide (0.3 vol.%)	0.04	7.04	2.21	82.42	0.42	1.78	0.02	nd	nd	nd	93.93
Residual liquid after removal of the phenocryst phases:											
Residual liquid composition[c]	52.75	0.40	11.51	2.95	0.06	1.11	2.44	3.17	1.65	0.18	76.23
Normalized[d]	69.20	0.52	15.10	3.88	0.08	1.46	3.21	4.16	2.16	0.23	100.00

Notes:

a: Median whole rock (WR) composition calculated from the 14 whole rock analyses reported in Table S3.

b: Median phenocryst compositions and proportion (in vol.%) of plagioclase, amphibole, orthopyroxene, and oxide phenocrysts. nd: no data, and present in negligible amount.

c: Residual liquid composition recalculated accounting for removal of the phenocryst phases from the normalized median whole rock composition relative to their proportions on a vesicle-free basis. For example, for residual SiO_2:

$$\text{Residual SiO}_2 = \text{WR}_{\text{SiO}_2} - (\text{vol.\%(pl)} \times \text{pl}_{\text{SiO}_2} + \text{vol.\%(amph)} \times \text{amph}_{\text{SiO}_2} + \text{vol.\%(opx)} \times \text{opx}_{\text{SiO}_2} + \text{vol.\%(ox)} \times \text{ox}_{\text{SiO}_2})/100$$

$$\text{Residual SiO}_2 = 65.56 - (17.9 \times 55.86 + 3.8 \times 45.01 + 2.0 \times 53.82 + 0.3 \times 0.04)/100$$

d: Normalized composition used in the GRD viscosity model.

Table S7 Results of the GRD viscosity models and final apparent dacite magma viscosity

T (°C)	820	900	820	900
H_2O (wt.%)	0.5		2.5	
A	-4.55		-4.55	
B	9631.6		9115.3	
C	311.2		194.3	
η_{melt} ($\log_{10}$ Pa.s)	7.77	6.62	5.59	4.76
η_{melt} (Pa.s)	5.89×10^7	4.17×10^6	3.89×10^5	5.75×10^4
η_{magma} ($\log_{10}$ Pa.s)	8.24	7.10	6.06	5.24
η_{magma} (Pa.s)	1.75×10^8	1.24×10^7	1.16×10^6	1.71×10^5

Notes:

- Temperature from the amphibole-only thermometer model of Ridolfi et al. (2010)
- Range of dissolved melt water content from the amphibole-only hygrometer of Ridolfi et al. (2010) minus ~4 wt.%.
- A, B, and C, are the GRD fitting parameters.
- η_{magma} calculated by multiplying the melt viscosity by the effect of suspended crystals (i.e., $\eta_r = 2.97$) using Equation 2.4 from Mueller et al. (2010).

References

Blundy, J., & Cashman, K. (2005). Rapid decompression-driven crystallization recorded by melt inclusions from Mount St. Helens volcano. Geology, *33*(10), 793-796. https://doi.org/10.1130/G21668.1

Chevrel, M. O., Platz, T., Hauber, E., Baratoux, D., Lavallée, Y., & Dingwell, D. B. (2013). Lava flow rheology: A comparison of morphological and petrological methods. Earth Planet Sci Lett, *384*, 109-120. https://doi.org/10.1016/j.epsl.2013.09.022

Fulcher, G. S. (1925). Analysis of recent measurements of the viscosity of glasses. J Am Ceram Soc, *8*(6), 339-355.

Giordano, D., Russell, J. K., & Dingwell, D. B. (2008). Viscosity of magmatic liquids: a model. Earth Planet Sci Lett, *4*, *271*(1-4), 123-134. https://doi.org/10.1016/j.epsl.2008.03.038

Krieger, I.M., & Dougherty, T.J. (1959). A Mechanism for Non-Newtonian Flow in Suspensions of Rigid Spheres. Trans. Soc. Rheol. *3*, 137–152. https://doi.org/10.1122/1.548848

Le Bas, M. L., Maitre, R. L., Streckeisen, A., Zanettin, B., & IUGS Subcommission on the Systematics of Igneous Rocks. (1986). A chemical classification of volcanic rocks based on the total alkali-silica diagram. J Petrol, *27*(3), 745-750. https://doi.org/10.1093/petrology/27.3.745

Mader, H. M., Llewellin, E. W., & Mueller, S. P. (2013). The rheology of two-phase magmas: A review and analysis. J Volcanol Geotherm Res *257*, 135-158. https://doi.org/10.1016/j.jvolgeores.2013.02.014

Maron, S. H., & Pierce, P. E. (1956). Application of ree-eyring generalized flow theory to suspensions of spherical particles. J Colloid Sci, *11*(1), 80-95. https://doi.org/10.1016/0095-8522(56)90023-X

Mueller, S., Llewellin, E. W., & Mader, H. M. (2010). The rheology of suspensions of solid particles. Proc R Soc A , *466*(2116), 1201-1228. https://doi.org/10.1098/rspa.2009.0445

Pinkerton, H., & Stevenson, R. J. (1992). Methods of determining the rheological properties of magmas at sub-liquidus temperatures. J Volcanol Geotherm Res, *53*(1-4), 47-66. https://doi.org/10.1016/0377-0273(92)90073-M

Ridolfi, F., Renzulli, A., & Puerini, M. (2010). Stability and chemical equilibrium of amphibole in calc-alkaline magmas: an overview, new thermobarometric formulations and application to subduction-related volcanoes. Contrib Mineral Petrol, *160*(1), 45–66. https://doi.org/10.1007/s00410-009-0465-7

Ridolfi, F. & Renzulli, A. (2012). Calcic amphiboles in calc-alkaline and alkaline magmas: thermobarometric and chemometric empirical equations valid up to 1,130° C and 2.2 GPa. Contrib Mineral Petrol, *163*(5), 877-895. https://doi.org/10.1007/s00410-011-0704-6

Ridolfi, F. (2021). Amp-TB2: an updated model for calcic amphibole thermobarometry. Minerals, *11*(3), 324. https://doi.org/10.3390/min11030324

Rutherford, M. J, & Hill, P. M. (1993). Magma ascent rates from amphibole breakdown: an experimental study applied to the 1980–1986 Mount St. Helens eruptions. J Geophys Res Solid Earth *98:19667–19685*. https://doi.org/10.1029/93JB01613

Rutherford, M. J,, Sigurdsson, H., Carey, S, & Davis, A. (1985). The May 18, 1980, eruption of Mount St. Helens: 1. melt composition and experimental phase equilibria. J Geophys Res Solid Earth *90:2929–2947*. https://doi.org/10.1029/JB090iB04p02929

Seligman, A. N,, Bindeman, I,, Van Eaton, A, & Hoblitt, R. (2018). Isotopic insights into the degassing and secondary hydration of volcanic glass from the 1980 eruptions of Mount St. Helens. Bull Volcanol *80:37*. https://doi.org/10.1007/s00445-018-1212-6

Schneider, C. A., Rasband, W. S., & Eliceiri, K. W. (2012). NIH Image to ImageJ: 25 years of image analysis. Nat Methods, *9*(7), 671-675. https://doi.org/10.1038/nmeth.2089

Tammann, G. H. W. Z., & Hesse, W. (1926). Die Abhängigkeit der Viscosität von der Temperatur bei unterkühlten Flüssigkeiten. *Z Anorg Allg Chem*, *156*(1), 245-257.

Vogel, D.H. (1921). Temperaturabhängigkeitsgesetz der Viskosität von Flüssigkeiten. Phys. Z., 22, 645–646.

SM3. Input parameters sampled for model runs

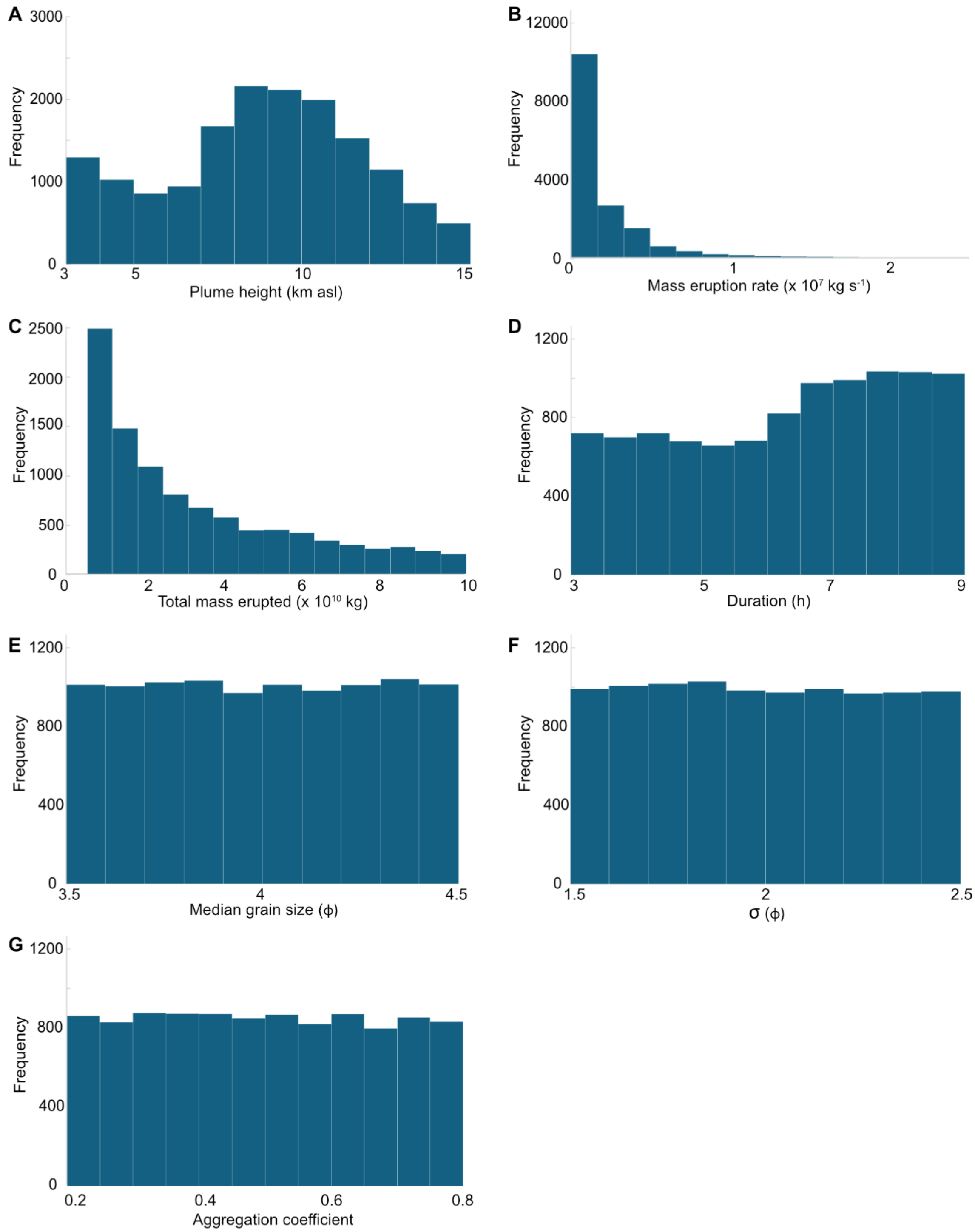

Figure S5 Input parameters sampled for VEI 3 eruption scenario (total of 10000 runs). Source parameters: **A**) plume height, **B**) mass eruption rate, **C**) total mass erupted, **D**) duration. Grain size parameters: **E**) median grain size, **F**) standard deviation of grain size (σ), **G**) aggregation coefficient. Note: plume height and mass eruption rate are sampled every 6 hours (resulting in 15857 selections for 10000 runs of the eruption lasting 3 – 9 hours)

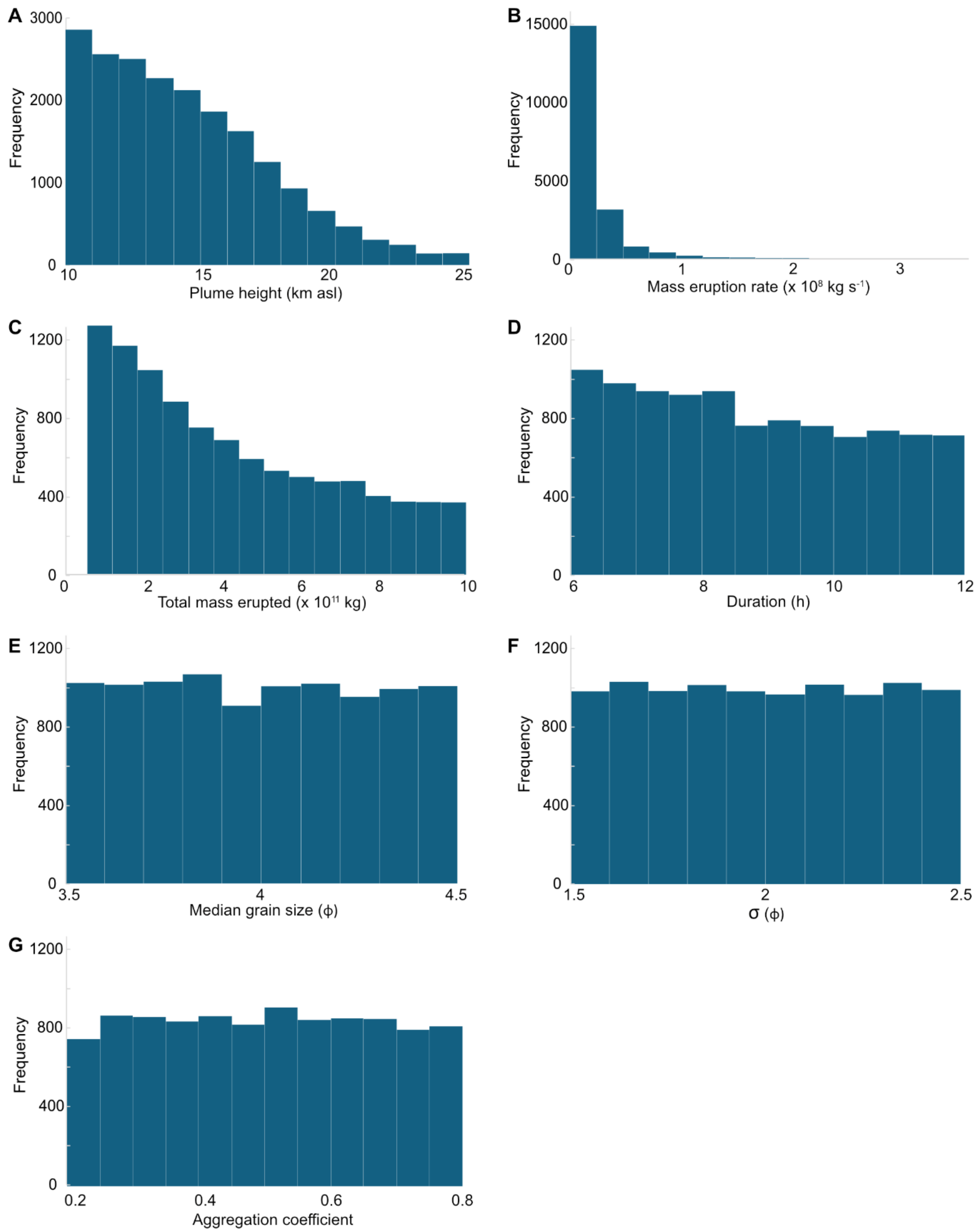

Figure S6 Input parameters sampled for VEI 4 eruption scenario (total of 10000 runs). Source parameters: **A**) plume height, **B**) mass eruption rate, **C**) total mass erupted, **D**) duration. Grain size parameters: **E**) median grain size, **F**) standard deviation of grain size (σ), **G**) aggregation coefficient. Note: plume height and mass eruption rate are sampled every 6 hours (resulting in 20000 selections for 10000 runs of the eruption lasting 6 – 12 hours)
