# Supplementary material for: Tephra fall probabilistic hazard maps for VEI 3 and VEI 4 eruptions at Mt. Garibaldi (Nch’kay’) volcano, British Columbia, Canada
Source: J Appl Volcanol. 2026 Sep 17;15(1):14. doi: 10.1186/s13617-026-00173-8 (PMC13582183; doi:10.1186/s13617-026-00173-8)
Supplement: Supplementary file 2 [file 13617_2026_173_MOESM2_ESM.pdf]

Tephra fall hazard at Mount Garibaldi (*Nch'kay'*) volcano, British Columbia, Canada

Natural Resources Canada / Ressources naturelles Canada
Lancaster University
UK Research and Innovation

Sara Osman, Thomas J. Jones, Yannick Le Moigne, Melanie Kelman

Volcanic hazard at Mount Garibaldi (*Nch'kay'*)

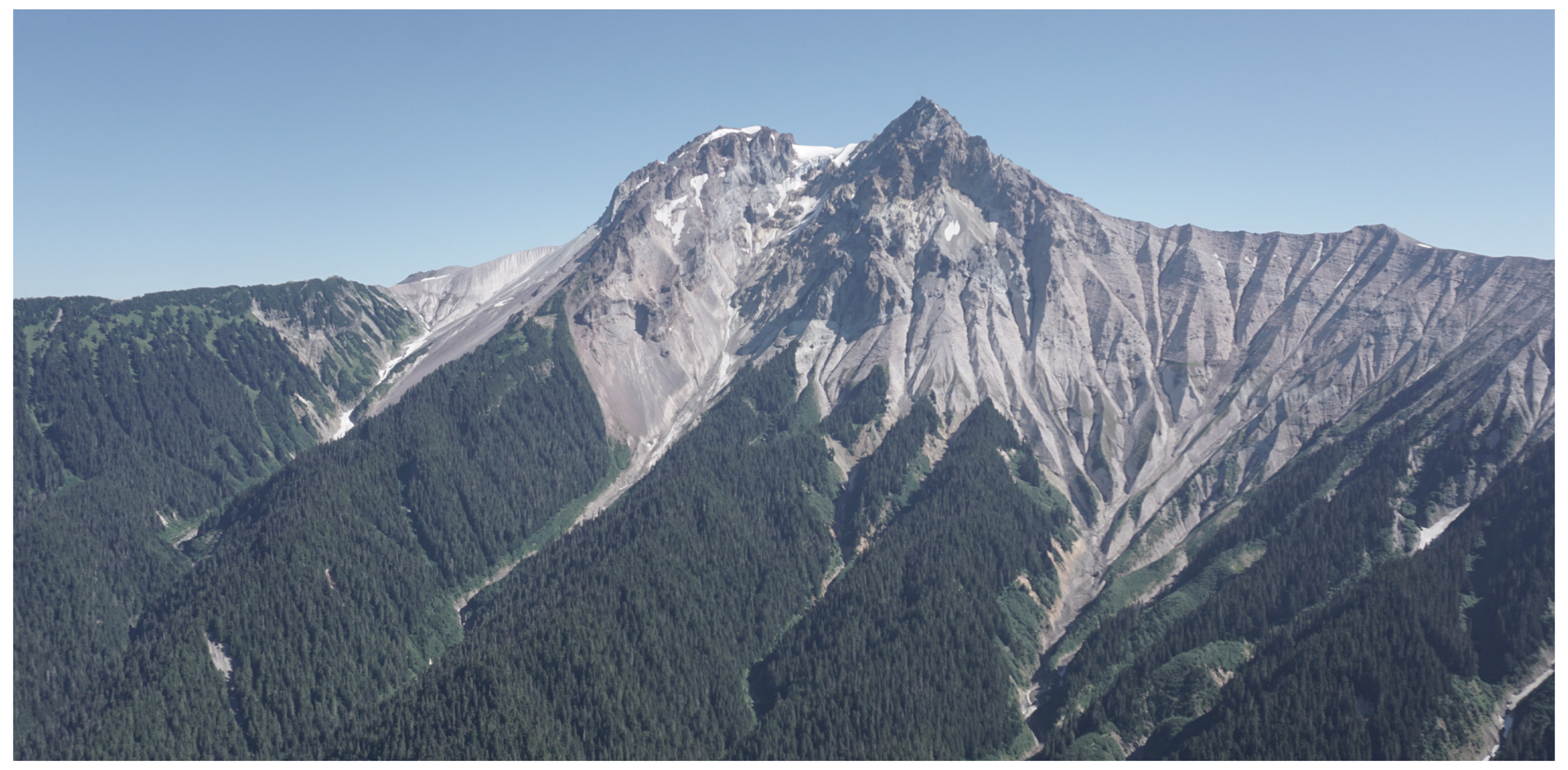

Mt. Garibaldi (*Nch'kay'*, 49.8506 °N, 123.0047 °W, 2,678 m asl) is the most conspicuous edifice of the Garibaldi – Price volcanic field (GPVF), situated approximately 60 km north of Vancouver, between the towns of Squamish and Whistler. The GPVF is part of the Garibaldi Volcanic Belt which consists of nine major volcanic fields from Glacier Peak in the south to Mt. Silverthrone in the north.

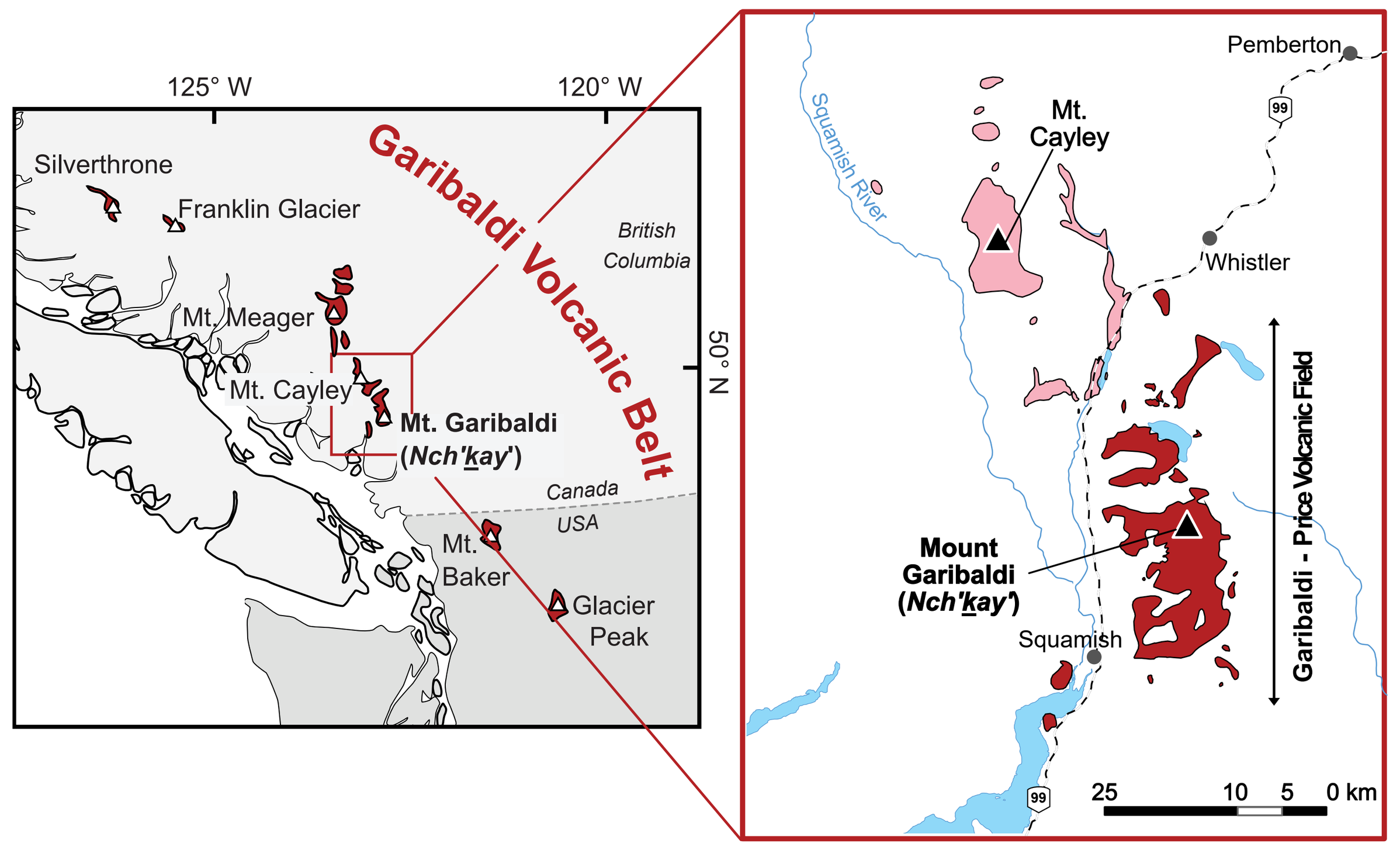

The geological history and geomorphology of Mt. Garibaldi are complex and intimately coupled with the advance and retreat of Cordilleran Ice. Mt. Garibaldi was intermittently active from the Middle Pleistocene to the Holocene with the last eruption occurring about 9.5 ka. Compositionally, the erupted lavas are dominantly dacitic in composition with evidence of effusive (domes, lava flows) and explosive eruptions.

Mt. Garibaldi has been assessed as a high-threat volcano, based on evidence of past eruptions and unrest, and risk to populations and infrastructure from a future eruption. It is in the same threat category as volcanoes such as Mt. St. Helens.

Probability of tephra load thresholds being exceeded

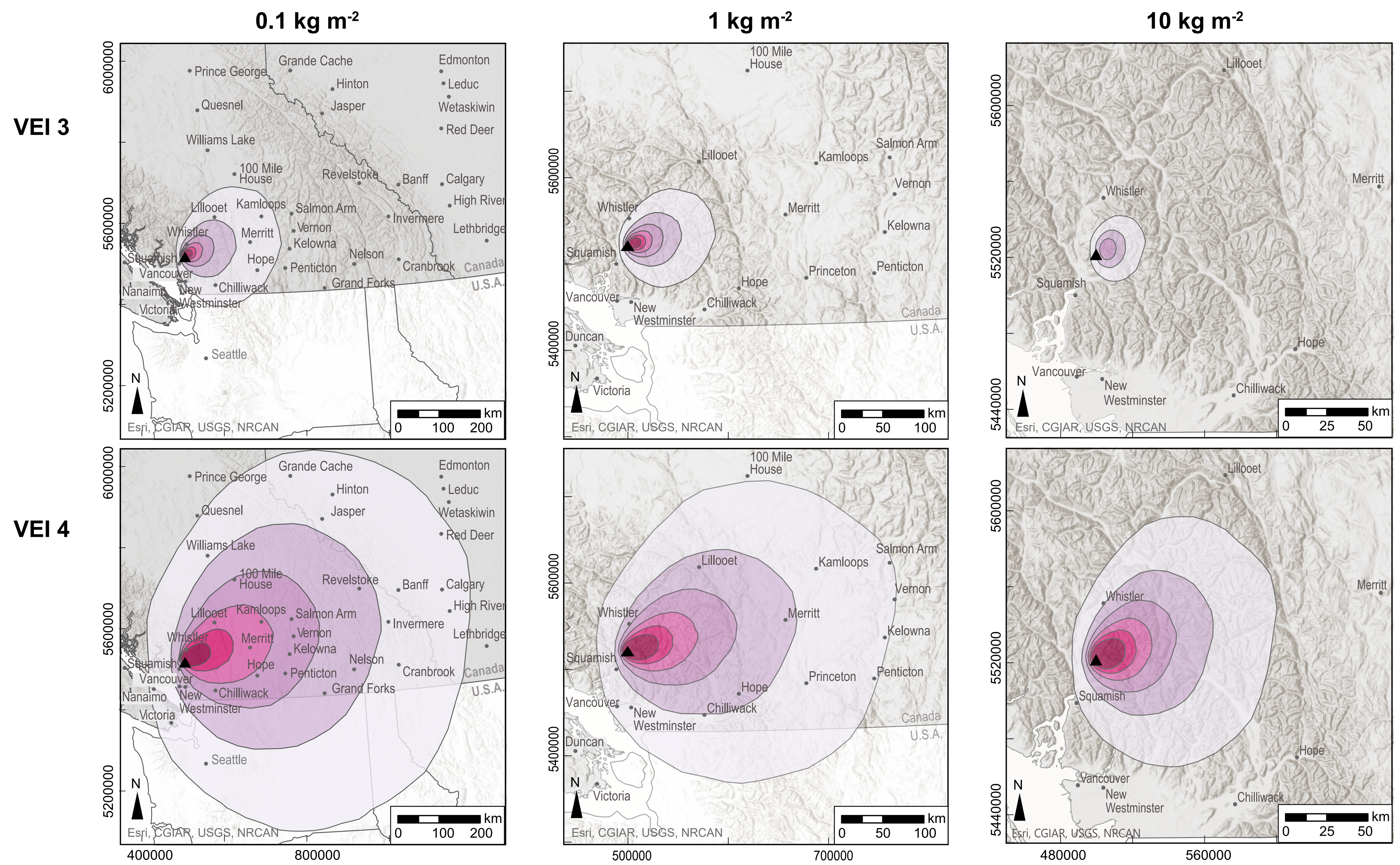

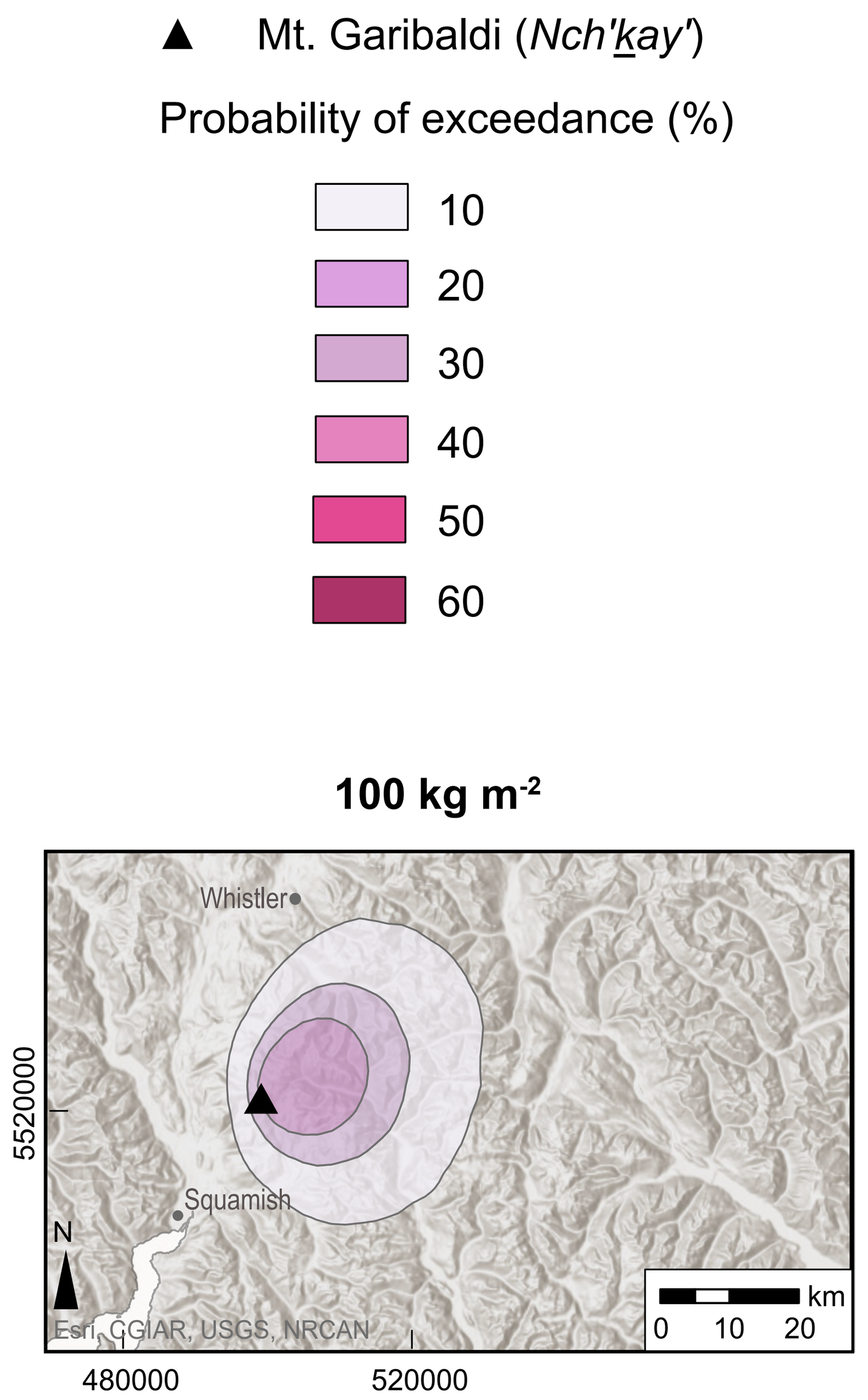

Loads of 100 kg m^{-2} only occur for the VEI 4 eruption scenario, and extend ~ 30 km from the volcano for a 10 % probability of exceedance.

Modelling tephra fall hazard at Mount Garibaldi (*Nch'kay'*)

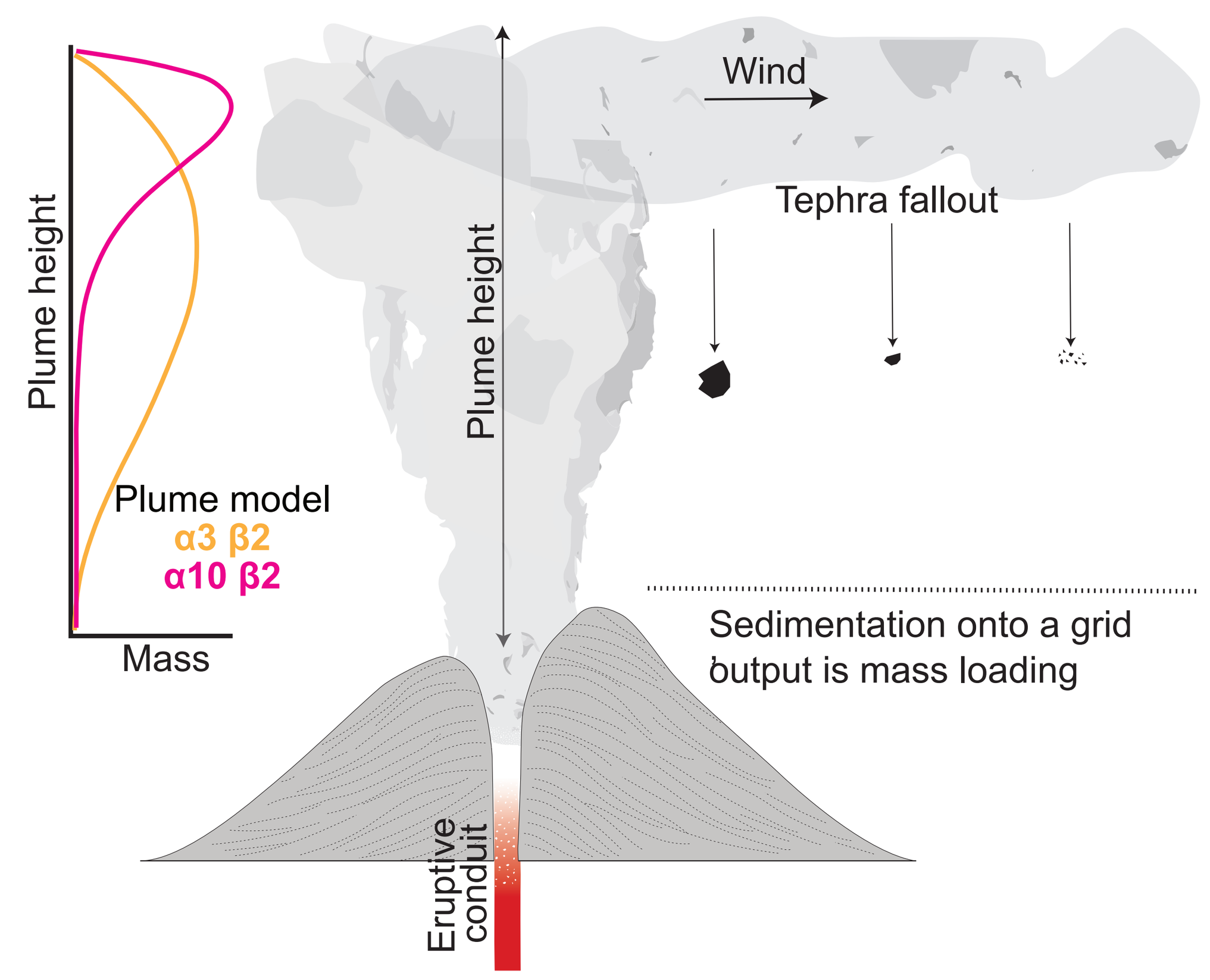

The Volcanic Explosivity Index (VEI) categorises the size of explosive eruptions based on the volume of ejecta and plume height, and evidence from previous eruptions suggests that VEI 4 and VEI 3 may occur at Mt. Garibaldi (*Nch'kay'*).

Eruption scenarios

VEI 3
Plume height 3 – 15 km
Duration 3 – 9 hours
Mass erupted 5 x10^{9} – 1 x10^{11} kg

VEI 4
Plume height 10 – 25 km
Duration 6 – 12 hours
Mass erupted 5 x 10^{10} – 1 x 10^{12} kg

By modelling these scenarios, we can identify areas that may be at risk of tephra fall during any future eruption. Results from our simulations show:

- probabilities of tephra thresholds being exceeded (top centre panel)
- tephra loads with 10, 50 and 90 % probability of being exceeded (bottom centre panel).

Tephra loads at fixed probability of being exceeded

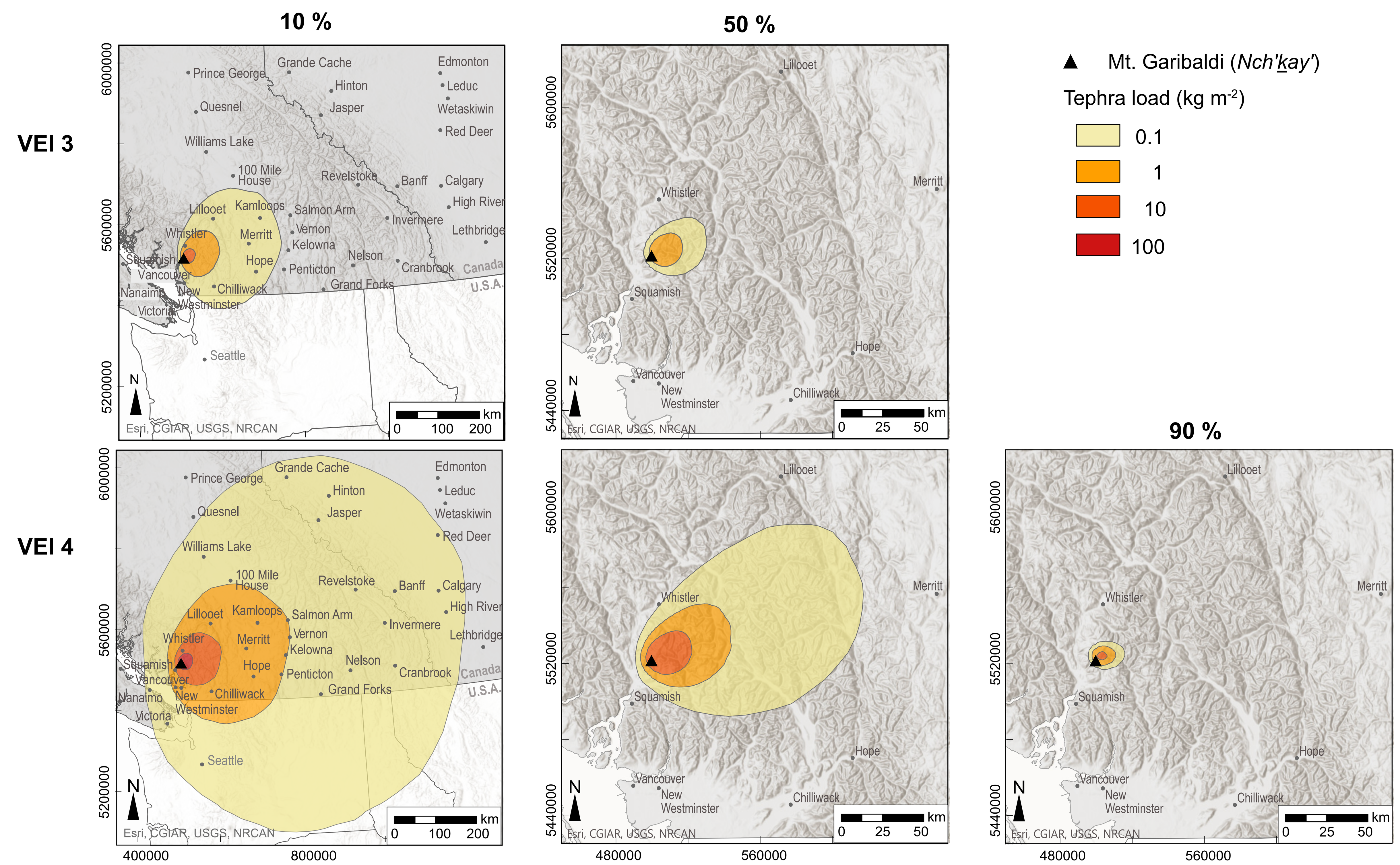

Tephra fall impacts

Impacts on infrastructure from tephra fall depend on the load of the deposit.

Tephra load (kg m^{-2})	Likely impact
0.1	Skid risks on roads, airports
1	Disruption to road, rail, airports<br>Clogging of heating, ventilation and air conditioning systems
10	Failure of power and telecomms cables<br>Flashover on high voltage lines
100	Collapse of weakest roofs and long span buildings

Area potentially impacted by tephra load thresholds for 10, 50 and 90 % probability of exceedance

Tephra load (kg m^{-2})	Total area impacted (km^2) VEI 3 10 %	VEI 3 50 %	VEI 4 10 %	VEI 4 50 %	VEI 4 90 %
0.1	56,010	810	572,820	9,360	230
1	8,190	250	79,840	1,670	100
10	820		11,470	450	20
100			1,160		

Acknowledgements: Funding was provided by a UKRI Future Leaders Fellowship (MR/W009781/1), Canada Safety and Security Program (CSSP-2022-CP-2537) and the associated Volcano Risk Reduction in Canada (VRRC) project.
